# Modulated sampled-data consensus for networked Euler-Lagrange systems with differentiable pulse function

**DOI:** 10.1371/journal.pone.0274461

**Published:** 2022-11-10

**Authors:** Yongzhi Sun, Yilin Wang, Yang Li, Xinjian Xiang

**Affiliations:** School of Automation and Electrical Engineering, Zhejiang University of Science & Technology, Hangzhou, China; University of Porto Faculty of Engineering: Universidade do Porto Faculdade de Engenharia, PORTUGAL

## Abstract

This paper is concerned with the sampled-data consensus of networked Euler-Lagrange systems. The Euler-Lagrange system has enormous advantages in analyzing and designing dynamical systems. Yet, some problems arise in the Euler-Lagrange equation-based control laws when they contain sampled-data feedbacks. The control law differentiates the discontinuous sampled-data signals to generate its control input. In this process, infinities in the control inputs are generated inevitably. The main goal of this work is to eliminate these infinities and make the control inputs applicable. To reach this goal, a class of differentiable pulse functions is designed for the controllers. The pulse functions work as multipliers on the sampled-data signals to make them differentiable, hence avoid the infinities. A new consensus condition compatible with the pulse function is also obtained through rigorous consensus analysis. The condition is proved to be less conservative compared with that of the existing method. Finally, numerical examples are given to illustrate the findings and theoretical results.

## Introduction

Multi-agent systems have become a popular research topic for more than a decade. The most fundamental and widely studied problem of multi-agent systems is the consensus problem, for it remains the most common control objective of multi-agent systems. And the studies such as formation control are derived from the consensus problem. A central concern of such studies is the dynamic models of the individual agents. For a theoretical formulation to properly reflect the actual physical system, the model has to be capable of representing the physical characteristics.

Euler-Lagrange system can describe a wide range of physical systems [1]. This advantage has inspired great research interest in Euler-Lagrange systems [2–9]. The theoretical progress has benefited the control of robotic arms [8], ships and robotic aerial vehicles [10], autonomous underwater vehicles [11], space crafts, and satellites [12, 13]. Yet much of the existing works on this subject assume continuous communication.

Discontinuities, e.g., event-triggering [14], constraints [15], and switching topology [16] are marking characteristics of modern networked systems. Above all, computer-based networked systems rely exclusively on sampled-data communication, the most common discontinuous information exchange. However, when sampled-data communication is applied to networked Euler-Lagrange systems [17], the control inputs go to infinity at the sampling instants, thus obstructing real-world implementations. The infinities are caused by differentiation of the discontinuous piecewise constant sampled communication information from the network. It is also worth mentioning that the Euler-Lagrange system with sampled actuation has already been studied in [18]. But without the distributed manner, there is no infinity problem.

Several works are dedicated to solving similar problems caused by discontinuities in networked Euler-Lagrange systems under continuous-time communication [19–22]. They all use high-order linear systems to smoothen the discontinuities in the neighbors’ information and avoid infinities. However, these methods all depend on exploiting real-time local information to guarantee consensus, which is uncommon in sampled-data systems.

Though the methods mentioned above can’t be used to solve the sampled-data consensus of networked Euler-Lagrange systems, the idea of smoothening the discontinuities is inspiring. In sampled-data systems, although the piecewise constant information is an unchangeable fundamental fact in all networked systems, changes can still be made within individual subsystems to work around. Conventional sampled-data controllers operate continuously with constant inputs during each sampling interval. This requirement may hinder controllers’ implementation under certain constraints [23]. Pulse-modulated sampled-data control is developed in [23, 24] that has advantages over the conventional sampled-data control: 1) the control input can be a time-varying during an interval; 2) the controller can work during part of the interval instead of the whole.

In this work, we take advantage of the pulse function, propose the differentiable pulse function, and position the discontinuous sampling instants in idle times to eliminate the infinities and make the controller applicable. A new consensus criterion is also obtained through rigorous and straightforward proof. It is proven that this criterion is less conservative and is compatible with the new pulse function proposed, whereas the existing one is not.

The rest of the paper is organized as follows. In Section II, preliminaries on the problem investigated are presented, and the problem of infinities is pointed out. Section III proposes constraints on the pulse function under which the infinities are ruled out. The new consensus criterion is given through consensus analysis and compared with the existing work in terms of conservativeness and applicability. Finally, numerical examples are provided in Section IV to verify the theoretical results.

## Preliminaries and problem formulation

### Graph theory

Communication links among the agents can be described by a weighted directed graph (digraph) $G=\left\{V,\;E,A\right\}$, where *V* = {1, 2, …, *N*} is the set of nodes, E=V×V is the set of edges, and *A* = (*a*_*ij*_)_(*N*×*N*)_ is the weighted adjacency matrix. {j,i}∈E indicates that agent *i* receives information from agent *j*. *a*_*ij*_ > 0 if and only if (j,i)∈E, otherwise *a*_*ij*_ > 0. Assume that there’s no self-loop, i.e. *a*_*ii*_ = 0, i∈V. Let $\text{deg}\left(i\right)=\sum_{j=1}^{N}a_{ij}$, *D* = *diag*(*deg*(1), …, *deg*(*n*)). The Laplacian matrix is *AL* = (*l*_*ij*_)_(*N*×*N*)_ = *D* − *A*. All eigenvalues of *L* are in the open right half plane except for the one zero eigenvalue: 0 = *λ*_1_ ≤ *Re*(*λ*_2_) ≤ ⋯ ≤ *Re*(*λ*_*r*_) where *λ*_*i*_ ∈ *C* (*i* = 1, 2, …, *r*) are eigenvalues of *L* with multiplicity *N*_*i*_. Obviously $\sum_{i}^{r}N_{i}=N$. There exists a non-singular matrix $U=\left(\frac{1_{N}}{\sqrt{N}},u_{2},\;\ldots ,\;u_{N}\right)$ such that the Laplacian matrix *L* can be transformed into a Jordan form

$$J=U^{-1}LU=diag\left\{0,\;J_{2},\;\ldots ,\;J_{r}\right\}$$

where $U^{-1}={\left(\sqrt{N}\xi ,\;w_{2},\;\ldots ,\;w_{N}\right)}^{T}$ and *ξ*^*T*^ *L* = 0, *ξ* is a vector such that $1_{N}^{T}\xi =1$.


$$J_{l}={\left[\begin{array}{ccc} \lambda_{l} & 1 & 0\\ 0 & \ddots & 1\\ 0 & 0 & \lambda_{l} \end{array}\right]}_{N_{l}\times N_{l}}$$

When the graph G is undirected, the Laplacian matrix *L* is symmetric and can be diagonalized.

### Networked Euler-Lagrange systems

Consider *N* networked Euler-Lagrange systems that are fully actuated and have the following dynamics:

$$M_{i}\left(q_{i}\right){\ddot{q}}_{i}+C_{i}\left(q_{i},{\dot{q}}_{i}\right){\dot{q}}_{i}+g_{i}\left(q_{i}\right)=\tau_{i},\;i=1,\;2,\;\ldots ,\;N$$ (1)

where $q_{i}={\left[q_{i1},\;q_{i2},\;\ldots ,\;q_{im}\right]}^{T}\in R^{m}$ is the generalized position, $M_{i}\left(q_{i}\right)=M_{i}^{T}\left(q_{i}\right)\in R^{m\times m\;}$ is the inertia matrix, $C_{i}\left(q_{i},{\dot{q}}_{i}\right)\in R^{m\times m\;}$ is the Coriolis and centripetal matrix, $g_{i}\left(q_{i}\right)\in R^{m}$ is the gravitational torque, $\tau_{i}=\in R^{m}$ is the control input, and the following general assumptions hold for the Euler-Lagrange system (1):

1. There exist positive-definite parameters *k*_*c*_ and *k*_*d*_ such that 0 < *k*_*c*_*I*_*m*_ ≤ *M*_*i*_ (*q*_*i*_) ≤ *k*_*d*_*I*_*m*_.
2. ${\dot{M}}_{i}\left(q_{i}\right)-2C_{i}\left(q_{i},{\dot{q}}_{i}\right)$ is skew-symmetric, i.e., for any $r\in R^{m},\;r^{T}\left({\dot{M}}_{i}\left(q_{i}\right)-2C_{i}\left(q_{i},{\dot{q}}_{i}\right)\right)r=0$.

#### Sampled-data consensus

The networked Euler-Lagrange systems in (1) are sampled at *t*_*k*_, *k* = 0, 1, …, where 0 = *t*_0_ < *t*_1_ < … < *t*_*k*_ < …, and *t*_*k*_ → ∞ as *k* → ∞. The sampling intervals can be time-varying: *h*_*k*_ = *t*_*k*+1_ − *t*_*k*_. The control objective is to design sampled-data consensus controllers that drive the networked Euler-Lagrange systems in (1) to achieve consensus, i.e., ∀i,j∈V:

$$\underset{t\to \infty }{\text{lim}}\left(q_{i}\left(\text{t}\right)-q_{j}\left(t\right)\right)=0$$

The following sampled-data consensus control law is designed in [17]:

$$\tau_{i}=-K_{i}s_{i}+M_{i}\left(q_{i}\right){\ddot{q}}_{r,i}+C_{i}\left(q_{i},{\dot{q}}_{i}\right){\dot{q}}_{r,i}+g_{i}\left(q_{i}\right)$$ (2)

where *K*_*i*_ is a positive-definite matrix. Eq 2 shows that the control law depends on the reference quantity ${\dot{q}}_{r,i}$ and its derivative ${\ddot{q}}_{r,i}$. And the reference quantity ${\dot{q}}_{r,i}$

$${\dot{q}}_{r,i}=-\rho \alpha \left(t,\;t_{k}\right)\underset{j\in N_{i}}{\sum }a_{ij}\left(q_{i}\left(t_{k}\right)-q_{j}\left(t_{k}\right)\right),\;t\in \left[t_{k},\;t_{k+1}\right)$$ (3)

is piecewise constant due to the sample-data communication. And $s_{i}={\dot{q}}_{i}-{\dot{q}}_{r,i}$ is the auxiliary variable, *α*(*t*, *t*_*k*_) is a pulse function:

$$\alpha \left(t,\;t_{k}\right)=\left\{\begin{array}{c} \hat{\alpha }\left(t,\;t_{k}\right),\;t\in \left[t_{k},\;t_{k}+d_{k}\right]\\ 0,\;t\in \left(t_{k}+d_{k},t_{k+1}\right] \end{array}\right.,$$ (4)

where $\hat{\alpha }\left(t,\;t_{k}\right)$ is the scaling function during dwell time whose value is allowed to be discontinuous. It is proved in [17] that the networked Euler-Lagrange systems (1) under the control input (2) reach consensus when a criterion on the communication graph and the pulse function *α*(*t*, *t*_*k*_) is satisfied.

Since the sampled local consensus error $\sum_{j\in N_{i}}a_{ij}\left(q_{i}\left(t_{k}\right)-q_{j}\left(t_{k}\right)\right)$ is updated at each sampling instant and is kept constant during the sampling intervals, the reference velocity ${\dot{q}}_{r,i}$ is discontinuous at the sampling instants *t*_*k*_ and the end of dwell times i.e. *t*_*k*_ + *d*_*k*_. Under this control strategy, the ${\ddot{q}}_{r,i}$ term in the controller (2) is the derivative of the discontinuous ${\dot{q}}_{r,i}$:

$${\ddot{q}}_{r,i}\left(t\right)=\frac{d\left(-\rho \alpha \left(t,\;t_{k}\right)\sum_{j\in N_{i}}a_{ij}\left(q_{i}\left(t_{k}\right)-q_{j}\left(t_{k}\right)\right)\right)}{dt}$$ (5)

thus is infinite at the sampling instants. Combining with that *M*_*i*_(*q*_*i*_) is positive definite, the term $M_{i}\left(q_{i}\right){\ddot{q}}_{r,i}$ is infinite and makes *τ*_*i*_ infinite at these instants.

### Problem formulation

Infinities in the control input make the controller impractical for implementation despite its mathematical correctness. Moreover, the consensus criterion in [17] is conservative and obtained through complicated derivation. Considering these drawbacks of [17], the goal of this work is to:

1. Find a method to eliminate the infinities in the control input.
2. Establish for this method a compatible and less conservative consensus criterion.

## Main results

To solve the problem formulated in Section II, we propose an improved control law that eliminates the infinities in the controller by redesigning the pulse function $\hat{\alpha }\left(t,\;t_{k}\right)$. It can be proved that given some additional conditions on $\hat{\alpha }\left(t,\;t_{k}\right)$, the control inputs *τ*_*i*_ no longer have the infinity behavior discussed above.

### Solution for the infinities

For ease of analysis, let function *α*(*t*) be the combination of the pulse functions *α*(*t*, *t*_*k*_):

$$\alpha \left(t\right)=\left\{\begin{array}{c} \alpha \left(t,\;t_{0}\right),\;t\in \left[t_{0},\;t_{1}\right)\\ \alpha \left(t,\;t_{1}\right),\;t\in \left[t_{1},\;t_{2}\right)\\ \vdots \end{array}\right.$$ (6)

*Lemma 1*: Control input (2) is finite and Lipschitz if the pulse function $\hat{\alpha }\left(t,\;t_{k}\right)$ is differentiable with a Lipschitz constant *l*, i.e.

$$\left|\hat{\alpha }\left(x,\;t_{k}\right)-\hat{\alpha }\left(y,\;t_{k}\right)\right|\leq l\left|x-y\right|\;\forall \;x,y\in \left[t_{k},\;t_{k}+d_{k}\right]$$ (7)

if the following hold:

$$\hat{\alpha }\left(t_{k},\;t_{k}\right)=\hat{\alpha }\left(t_{k},\;t_{k}+d_{k}\right)=0$$ (8)

$$\underset{t\to t_{k}^{+}}{\text{lim}}\;\dot{\alpha }\left(t,\;t_{k}\right)=\underset{t\to {\left(t_{k}+d_{k}\right)}^{-}}{\text{lim}}\;\dot{\alpha }\left(t,\;t_{k}\right)=0,\;\;k=0,\;1,\;\ldots$$ (9)

*Proof*: It’s obvious that the pulse function *α*(*t*) is Lipschitz and differentiable when (8) and (9) hold. Then we prove the differentiability of ${\dot{q}}_{r,i}$ at the endpoints of the dwell time, i.e. *t*_*k*_ and *t*_*k*_ + *d*_*k*_.

Under condition (9), the left derivatives and right derivatives of ${\dot{q}}_{r,i}$ are

$$\underset{t\to t_{k}^{+}}{\text{lim}}\;{\ddot{q}}_{r,i}\left(t\right)=-\rho \;\underset{t\to t_{k}^{+}}{\text{lim}}\;\dot{\alpha }\left(t,\;t_{k}\right)\underset{j\in N_{i}}{\sum }a_{ij}\left(q_{i}\left(t_{k}\right)-q_{j}\left(t_{k}\right)\right)=0$$

$$\underset{t\to t_{k}^{-}}{\text{lim}}\;{\ddot{q}}_{r,i}\left(t\right)=-\rho \;\underset{t\to t_{k}^{-}}{\text{lim}}\;\dot{\alpha }\left(t,\;t_{k-1}\right)\underset{j\in N_{i}}{\sum }a_{ij}\left(q_{i}\left(t_{k-1}\right)-q_{j}\left(t_{k-1}\right)\right)=0$$

and

$$\underset{t\to {\left(t_{k}+d_{k}\right)}^{-}}{\text{lim}}\;{\ddot{q}}_{r,i}\left(t\right)=-\rho \;\underset{t\to {\left(t_{k}+d_{k}\right)}^{-}}{\text{lim}}\;\dot{\alpha }\left(t,\;t_{k}\right)\underset{j\in N_{i}}{\sum }a_{ij}\left(q_{i}\left(t_{k}\right)-q_{j}\left(t_{k}\right)\right)=0$$

$$\underset{t\to {\left(t_{k}+d_{k}\right)}^{+}}{\text{lim}}\;{\ddot{q}}_{r,i}\left(t\right)=-\rho \;\underset{t\to {\left(t_{k}+d_{k}\right)}^{+}}{\text{lim}}\;\dot{\alpha }\left(t,\;t_{k}\right)\underset{j\in N_{i}}{\sum }a_{ij}\left(q_{i}\left(t_{k}\right)-q_{j}\left(t_{k}\right)\right)=0$$

Note here that ${\dot{q}}_{r,i}$ is constantly zero out of dwell times, i.e. ${\dot{q}}_{r,i}\left(t\right)=0,\;t\in \left[t_{k}+d_{k},\;t_{k+1}\right]$, and the above analysis ensures ${\ddot{q}}_{r,i}\left(t\right)=0,\;t\in \left[t_{k}+d_{k},\;t_{k+1}\right]$. Therefore, ${\ddot{q}}_{r,i}\left(t_{k}\right)$ and ${\ddot{q}}_{r,i}\left(t_{k}+d_{k}\right)$ exists and are zero. Combined with the given condition that $\hat{\alpha }\left(t,\;t_{k}\right)$ is differentiable, ${\dot{q}}_{r,i}\left(t\right)$ is also differentiable during dwell times. Thus ${\dot{q}}_{r,i}$ is differentiable at *t* ∈ (0, ∞), therefore, ${\ddot{q}}_{r,i}$ is Lipschitz and finite with

$${\ddot{q}}_{r,i}\left(t\right)\leq l\underset{j\in N_{i}}{\sum }a_{ij}\left(q_{i}\left(t_{k}\right)-q_{j}\left(t_{k}\right)\right).$$

Applying controller (2) to the Euler-Lagrange system (1) yields

$$M_{i}\left(q_{i}\right){\dot{s}}_{i}+C_{i}\left(q_{i},{\dot{q}}_{i}\right)s_{i}=-K_{i}s_{i},\;i=1,\;2,\;\ldots ,\;N$$ (10)

Choose the Lyapunov function:

$$V\left(t\right)=\frac{1}{2}\overset{N}{\underset{i=1}{\sum }}s_{i}^{T}\left(t\right)M_{i}\left(q_{i}\right)s_{i}\left(t\right)$$ (11)

Its derivative along the trajectory of (10) is

$$\begin{array}{ll} \dot{V}\left(t\right) & =\frac{1}{2}\overset{N}{\underset{i=1}{\sum }}({\dot{s}}_{i}^{T}\left(t\right)M_{i}\left(q_{i}\right)s_{i}\left(t\right)+s_{i}^{T}\left(t\right)M_{i}\left(q_{i}\right){\dot{s}}_{i}\left(t\right)+s_{i}^{T}\left(t\right){\dot{M}}_{i}\left(q_{i}\right)s_{i}\left(t\right))\\ & =\frac{1}{2}\overset{N}{\underset{i=1}{\sum }}\left(-2s_{i}^{T}C_{i}\left(q_{i},{\dot{q}}_{i}\right)s_{i}-2s_{i}^{T}K_{i}s_{i}+s_{i}^{T}\left(t\right){\dot{M}}_{i}\left(q_{i}\right)s_{i}\left(t\right)\right)\\ & =-\overset{N}{\underset{i=1}{\sum }}s_{i}^{T}K_{i}s_{i}<0 \end{array}$$ (12)

Note here that inequality (12) holds on all *t* ≥ 0, instead of just sampling intervals. This is attributed to the controller design (2) and the subsequent closed-loop dynamics (10). Therefore, *s*_*i*_ → 0 as *t* → ∞. Combine (3) with the auxiliary variable *s* yields

$${\dot{q}}_{i}=s_{i}+{\dot{q}}_{r,i}=s_{i}-\rho \alpha \left(t\right)\underset{j\in N_{i}}{\sum }a_{ij}\left(q_{i}\left(t_{k}\right)-q_{j}\left(t_{k}\right)\right)$$ (13)

Eq (13) can be represented in stack vector form:

$$\dot{q}={\dot{q}}_{r}+s=-\rho \alpha \left(t\right)\left(L⊗I_{m}\right)q\left(t_{k}\right)+s$$ (14)

where *q* and *s* are the stacked vectors for *q*_*i*_ and *s*_*i*_, respectively. Since *s* vanishes with time, the stability of the dynamic Eq (14) is equivalent to that of

$$\dot{q}=-\rho \alpha \left(t\right)\left(L⊗I_{m}\right)q\left(t_{k}\right)$$ (15)

Next, the consensus of system (15) is studied, and a consensus criterion is obtained.

### Consensus analysis

In the following theorem, the consensus of system (15) is studied, and a consensus criterion compatible with the new pulse function *α* is proposed.

*Theorem 1*: The multi-agent system composed of Euler–Lagrange systems in (1) with the control inputs (2) can reach consensus if the following inequality holds for *l* = 2, 3, …, *r* and *k* = 0, 1, …

$$\left|1-\rho \lambda_{l}\overset{t_{k+1}}{\underset{t_{k}}{\int }}\alpha \left(\tau ,t_{k}\right)dt\right|<1$$ (16)

*Proof*: Solve the dynamic Eq (15). One has

$$q\left(t\right)=q\left(t_{k}\right)+\int_{t_{k}}^{t}-\rho \alpha \left(\tau \right)\left(L⊗I_{m}\right)q\left(t_{k}\right)d\tau +\int_{t_{k}}^{t}s\left(t\right)d\tau$$ (17)

and

$$\begin{array}{c} q\left(t_{k+1}\right)=q\left(t_{k}\right)-\rho \left(L⊗I_{m}\right)q\left(t_{k}\right)\int_{t_{k}}^{t_{k+1}}\alpha \left(\tau ,\;t_{k}\right)d\tau \\ +\int_{t_{k}}^{t_{k+1}}s\left(t\right)d\tau \\ =\left(I_{Nm}-\rho \left(L⊗I_{m}\right)\int_{t_{k}}^{t_{k+1}}\alpha \left(\tau ,\;t_{k}\right)d\tau \right)q\left(t_{k}\right)\\ +\int_{t_{k}}^{t_{k+1}}s\left(t\right)d\tau \end{array}$$ (18)

Let the consensus error be ${\hat{q}}_{i}=q_{i}-\xi^{T}q$, and $\hat{q}={\left({\hat{q}}_{1}^{T},\;{\hat{q}}_{2}^{T},\;\ldots ,\;{\hat{q}}_{N}^{T}\right)}^{T}$. Then we have $\hat{q}=\left(\left(I_{N}-1_{N}\xi^{T}\right)⊗I_{m}\right)q$. Applying this transformation on (18) yields

$$\begin{array}{c} \hat{q}\left(t_{k+1}\right)=\left(\left(I_{N}-1_{N}\xi^{T}\right)⊗I_{m}\right)\times \\ \left(I_{Nm}-\rho \left(L⊗I_{m}\right)\int_{t_{k}}^{t_{k+1}}\alpha \left(\tau ,\;t_{k}\right)d\tau \right)q\left(t_{k}\right)\\ +\left(\left(I_{N}-1_{N}\xi^{T}\right)⊗I_{m}\right)\int_{t_{k}}^{t_{k+1}}s\left(t\right)d\tau \\ =\left(I_{Nm}-\rho \left(L⊗I_{m}\right)\int_{t_{k}}^{t_{k+1}}\alpha \left(\tau ,\;t_{k}\right)d\tau \right)\times \\ \left(\left(I_{N}-1_{N}\xi^{T}\right)⊗I_{m}\right)q\left(t_{k}\right)\\ +\left(\left(I_{N}-1_{N}\xi^{T}\right)⊗I_{m}\right)\int_{t_{k}}^{t_{k+1}}s\left(t\right)d\tau \\ =\left(I_{Nm}-\rho \left(L⊗I_{m}\right)\int_{t_{k}}^{t_{k+1}}\alpha \left(\tau ,\;t_{k}\right)d\tau \right)\hat{q}\left(t_{k}\right)\\ +\left(\left(I_{N}-1_{N}\xi^{T}\right)⊗I_{m}\right)\int_{t_{k}}^{t_{k+1}}s\left(t\right)d\tau \end{array}$$ (19)

The facts that *L*1_*N*_ = 0 and *ξ*^*T*^ *L* = 0 are exploited to obtain (19).

Let $y=\left(U^{-1}⊗I_{m}\right)\hat{q}={\left(y_{1}^{T},\;y_{2}^{T},\;\ldots ,\;y_{r}^{T}\right)}^{T},\;y_{l}\in R^{mN_{l}}$, *l* = 1, 2, …, *r*, $\hat{y}={\left(y_{2}^{T},\;\ldots ,\;y_{r}^{T}\right)}^{T}$. We get

$$\begin{array}{c} y\left(k+1\right)=\left(U^{-1}⊗I_{m}\right)\times \\ \left(I_{Nm}-\rho \left(L⊗I_{m}\right)\int_{t_{k}}^{t_{k+1}}\alpha \left(\tau ,\;t_{k}\right)d\tau \right)\left(U⊗I_{m}\right)y\left(t_{k}\right)\\ +\left(U^{-1}\left(I_{N}-1_{N}\xi^{T}\right)⊗I_{m}\right)\int_{t_{k}}^{t_{k+1}}s\left(t\right)d\tau \\ =\left(I_{Nm}-\rho \left(J⊗I_{m}\right)\int_{t_{k}}^{t_{k+1}}\alpha \left(\tau ,\;t_{k}\right)d\tau \right)y\\ +\left(U^{-1}\left(I_{N}-1_{N}\xi^{T}\right)⊗I_{m}\right)\int_{t_{k}}^{t_{k+1}}s\left(t\right)d\tau \\ =diag\left\{I_{m},\;{\hat{J}}_{2},\;{\hat{J}}_{3},\;\ldots ,\;{\hat{J}}_{r}\right\}y+\\ \left(U^{-1}\left(I_{N}-1_{N}\xi^{T}\right)⊗I_{m}\right)\int_{t_{k}}^{t_{k+1}}s\left(t\right)d\tau \end{array}$$ (20)

where ${\hat{J}}_{l}=I_{N_{l}m}-\rho \left(J_{l}⊗I_{m}\right)\int_{t_{k}}^{t_{k+1}}\alpha \left(\tau ,\;t_{k}\right)d\tau$, *l* = 2, 3, …, *r*. Note that

$$\begin{array}{c} y_{1}=\left(\sqrt{N}\xi^{T}⊗I_{m}\right)\hat{q}\\ =\left(\sqrt{N}\xi^{T}⊗I_{m}\right)\left(\left(I_{N}-1_{N}\xi^{T}\right)⊗I_{m}\right)q\\ =\sqrt{N}\left(\left(\xi^{T}-\xi^{T}1_{N}\xi^{T}\right)⊗I_{m}\right)q=0 \end{array}$$ (21)

which means *y*_1_ stays constantly zero. It can be deduced from (19) and (20) that

$$\begin{array}{c} \hat{y}\left(k+1\right)=diag\left\{{\hat{J}}_{2},\;{\hat{J}}_{3},\;\ldots ,\;{\hat{J}}_{r}\right\}\hat{y}\left(k\right)\\ +\left(U^{-1}\left(2:N\right)\left(I_{N}-1_{N}\xi^{T}\right)⊗I_{m}\right)\int_{t_{k}}^{t_{k+1}}s\left(t\right)d\tau \end{array}$$ (22)

Since $\int_{t_{k}}^{t_{k+1}}s\left(t\right)d\tau \to 0$ as *k* → ∞ and matrix *U* is non-singular, $\hat{q}\left(k\right)$ converges to zero if and only if $\hat{y}$ converges to zero. Eq (22) shows that ${\text{lim}}_{k\to \infty }\hat{y}\left(k\right)=0$ if the spectral radii $\rho \left(\;{\hat{J}}_{l}\right)<1$ for *l* = 2, 3, …, *r*. Eigenvalues of ${\hat{J}}_{l}$ is determined by its diagonal entries, which are

$$1-\rho \lambda_{l}\overset{t_{k+1}}{\underset{t_{k}}{\int }}\alpha \left(\tau ,t_{k}\right)dt,\;l=2,3,\ldots ,\;r$$

so it can be concluded that the closed-loop Euler–Lagrange systems can reach consensus if (16) holds.

### Comparison between the consensus criterions

The geometric interpretation of our improved criterion (16) in the complex plane is rather classical, that

$$1-\rho \lambda_{l}\overset{t_{k+1}}{\underset{t_{k}}{\int }}\alpha \left(\tau ,t_{k}\right)dt$$

is within the unit disc.

**Fig 1:**
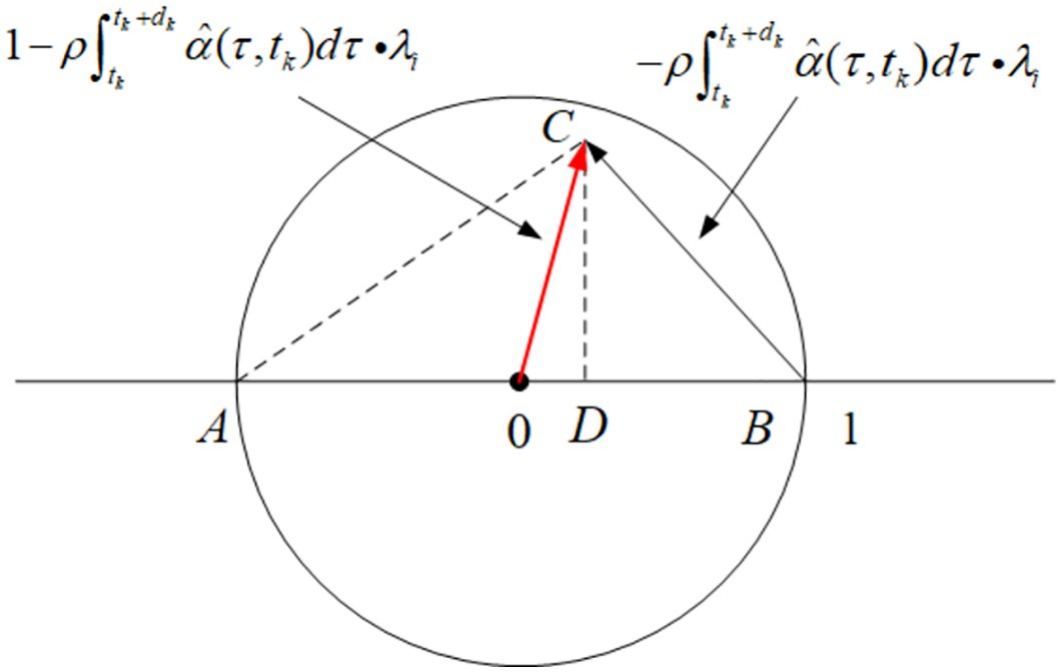
The geometric interpretation.

As is shown in Fig 1, the lengths of the line segments are:

$$\left|BD\right|=\rho \overset{t_{k+1}}{\underset{t_{k}}{\int }}\alpha \left(\tau ,t_{k}\right)dtRe\left(\lambda_{i}\right)$$

$$\left|AD\right|=2-\rho \overset{t_{k+1}}{\underset{t_{k}}{\int }}\alpha \left(\tau ,t_{k}\right)dtRe\left(\lambda_{i}\right)$$

$$\left|CD\right|=\rho \overset{t_{k+1}}{\underset{t_{k}}{\int }}\alpha \left(\tau ,t_{k}\right)dt\left|Im\left(\lambda_{i}\right)\right|$$

Point *C* is within the unit disk if and only if |*CD*|^2^ < |*AD*| ⋅ |*BD*|. By this geometric interpretation the criterion under which $1-\rho \lambda_{l}\int_{t_{k}}^{t_{k+1}}\alpha \left(\tau ,t_{k}\right)dt$ lies within the unit disk can be rewritten as

$${\left(\rho \overset{t_{k+1}}{\underset{t_{k}}{\int }}\alpha \left(\tau ,t_{k}\right)dt\left|Im\left(\lambda_{i}\right)\right|\right)}^{2}<\rho \overset{t_{k+1}}{\underset{t_{k}}{\int }}\alpha \left(\tau ,t_{k}\right)dtRe\left(\lambda_{i}\right)\left(2-\rho \overset{t_{k+1}}{\underset{t_{k}}{\int }}\alpha \left(\tau ,t_{k}\right)dtRe\left(\lambda_{i}\right)\right)$$

Singling out the $\rho \int_{t_{k}}^{t_{k+1}}\alpha \left(\tau ,t_{k}\right)dt$ term, the above inequality becomes

$$\rho \overset{t_{k+1}}{\underset{t_{k}}{\int }}\alpha \left(\tau ,t_{k}\right)dt<\frac{2Re\left(\lambda_{i}\right)}{{\left(Re\left(\lambda_{i}\right)\right)}^{2}+{\left(Im\left(\lambda_{i}\right)\right)}^{2}}$$ (23)

Next our new consensus criterion (16) is compared with the consensus criterion of [17]:

$$d\beta_{2}^{2}\rho {\left(Re\left(\lambda_{i}\right)+\left|Im\left(\lambda_{i}\right)\right|\right)}^{2}-\beta_{1}Re\left(\lambda_{i}\right)<0$$ (24)

where *d* is the upper bound of *d*_*k*_, *β*_1_ and *β*_2_ are the lower and upper bounds of the pulse function $\hat{\alpha }$, respectively. It can be inferred that the minimum lower bound of $\hat{\alpha }\left(t,\;t_{k}\right)$, i.e. *β*_1_ is positive, so we can divide (24) by *β*_1_(*Re*(*λ*_*i*_) + |*Im*(*λ*_*i*_)|)^2^ and get

$$\frac{\beta_{2}^{2}}{\beta_{1}}\rho d-\frac{Re\left(\lambda_{i}\right)}{{\left(Re\left(\lambda_{i}\right)+\left|Im\left(\lambda_{i}\right)\right|\right)}^{2}}<0$$

For every *λ*_*i*_, criterion (24) is equivalent to

$$\frac{\beta_{2}}{\beta_{1}}d\rho \beta_{2}<\frac{Re\left(\lambda_{i}\right)}{{\left(Re\left(\lambda_{i}\right)+\left|Im\left(\lambda_{i}\right)\right|\right)}^{2}}$$ (25)

It is intuitive to see that *dβ*_2_ is analogous to the term $\int_{t_{k}}^{t_{k+1}}\alpha \left(\tau ,t_{k}\right)dt$ as the area under the image of *α*(*τ*, *t*_*k*_) with the relation

$$\overset{t_{k+1}}{\underset{t_{k}}{\int }}\alpha \left(\tau ,t_{k}\right)dt<d\beta_{2}$$

Then, the comparison between criteria (16) and (24) is turned into that between (23) and (25). Comparing the left-hand sides, we get

$$\rho \overset{t_{k+1}}{\underset{t_{k}}{\int }}\alpha \left(\tau ,t_{k}\right)dt<\frac{\beta_{2}}{\beta_{1}}d\rho \beta_{2}$$

comparing the right-hand sides yields

$$\frac{Re\left(\lambda_{i}\right)}{{\left(Re\left(\lambda_{i}\right)+\left|Im\left(\lambda_{i}\right)\right|\right)}^{2}}<\frac{2Re\left(\lambda_{i}\right)}{{\left(Re\left(\lambda_{i}\right)\right)}^{2}+{\left(Im\left(\lambda_{i}\right)\right)}^{2}}$$

Therefore,

$$\rho \overset{t_{k+1}}{\underset{t_{k}}{\int }}\alpha \left(\tau ,t_{k}\right)dt<\frac{\beta_{2}}{\beta_{1}}d\rho \beta_{2}<\frac{Re\left(\lambda_{i}\right)}{{\left(Re\left(\lambda_{i}\right)+\left|Im\left(\lambda_{i}\right)\right|\right)}^{2}}<\frac{2Re\left(\lambda_{i}\right)}{{\left(Re\left(\lambda_{i}\right)\right)}^{2}+{\left(Im\left(\lambda_{i}\right)\right)}^{2}}$$

In other words, criterion (16) is a necessary condition of (24). This means when constructing a pulse function, one that satisfies (24) also satisfies (16), while one that meets (16) doesn’t necessarily satisfy (24). This implies that the range of selection of pulse functions under (16) is wider than that under (24), i.e. the improved criterion (16) is less conservative compared to (24).

*Remark 1*: A less conservative criterion gives an advantage when choosing the pulse function. One has wider choices with a less conservative criterion. The extreme case is the pulse function that eliminates infinities, which should be differentiable everywhere and vanishes at *t*_*k*_ and *t*_*k*_ + *d*_*k*_, which means its lower bound *β*_1_ = 0. This makes the left-hand side of (24) always positive, thus creating a contradiction and making (24) inapplicable. While this pulse function is compatible with (16). Such an example is included in the next section.

## Numerical examples

In this section, we first illustrate the infinities in the control inputs with the example in [17], then present a simulation with the differentiable pulse function that eliminates the infinities. In addition, a comparison between different pulse functions is provided to illustrate the effects of pulse functions on consensus speed.

### Simulation for [17]

**Fig 2:**
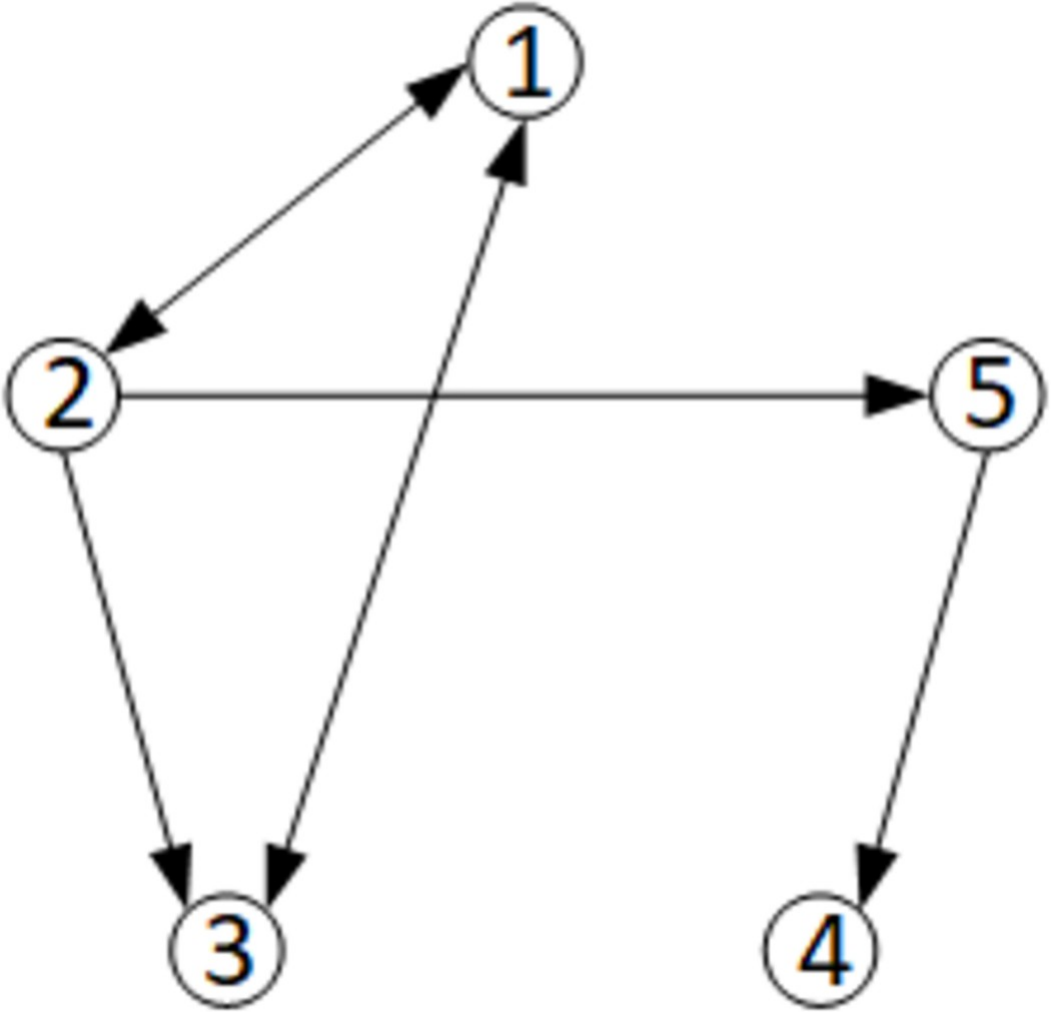
The communication graph.

Consider the closed-loop networked Euler-Lagrange Systems. Choose the same network as in Section IV of [17] with five agents, as shown in Fig 2:

The sampled-data communication is periodic with a sampling interval *h* = 1.

**Fig 3:**
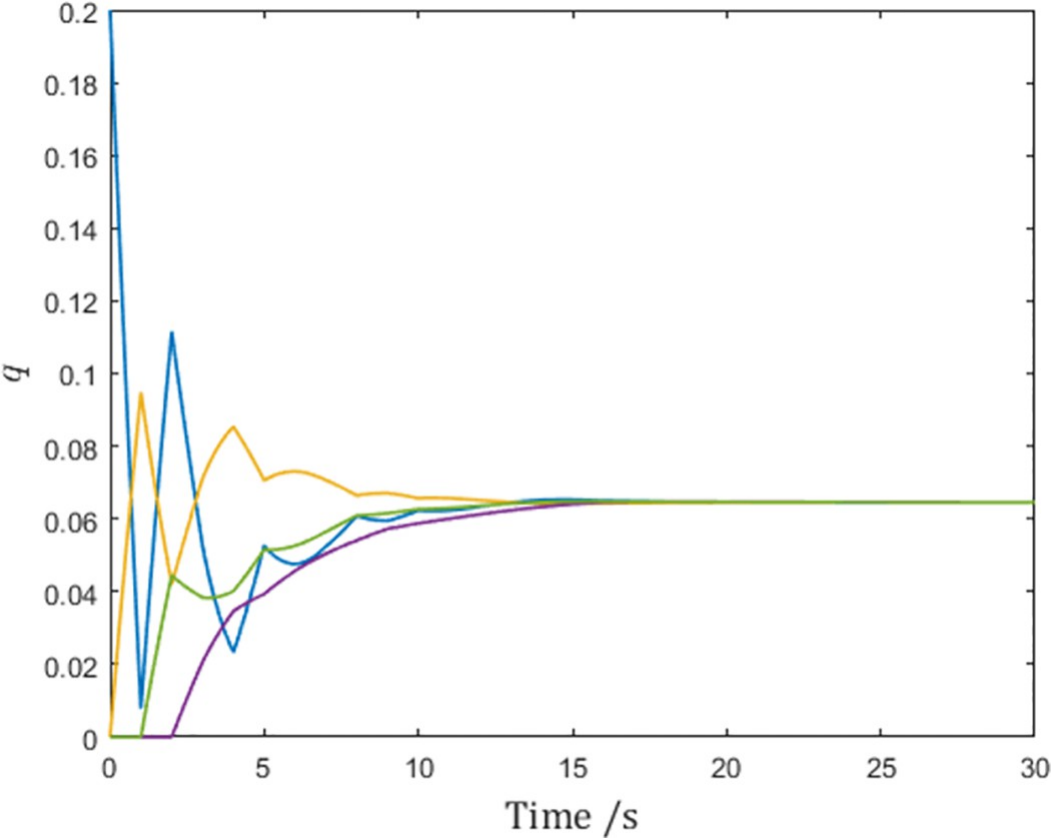
The generalized positions of the system.

**Fig 4:**
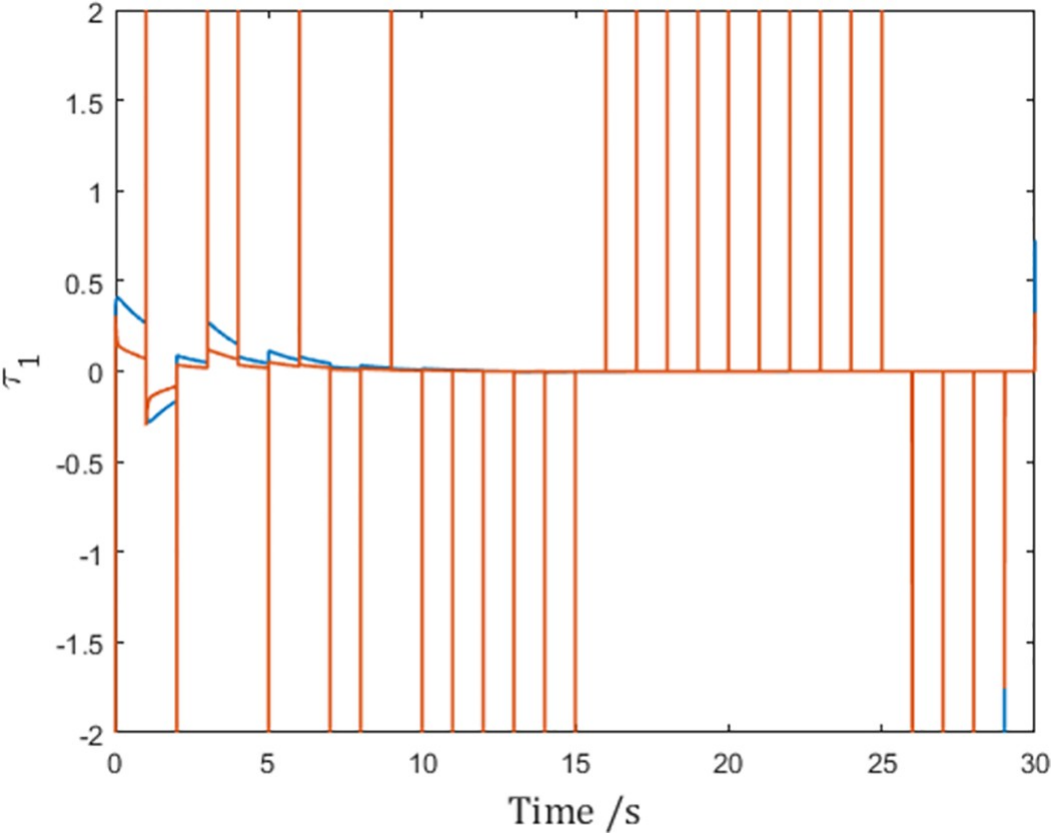
The control input *τ*_1_ of Agent 1.

**Fig 5:**
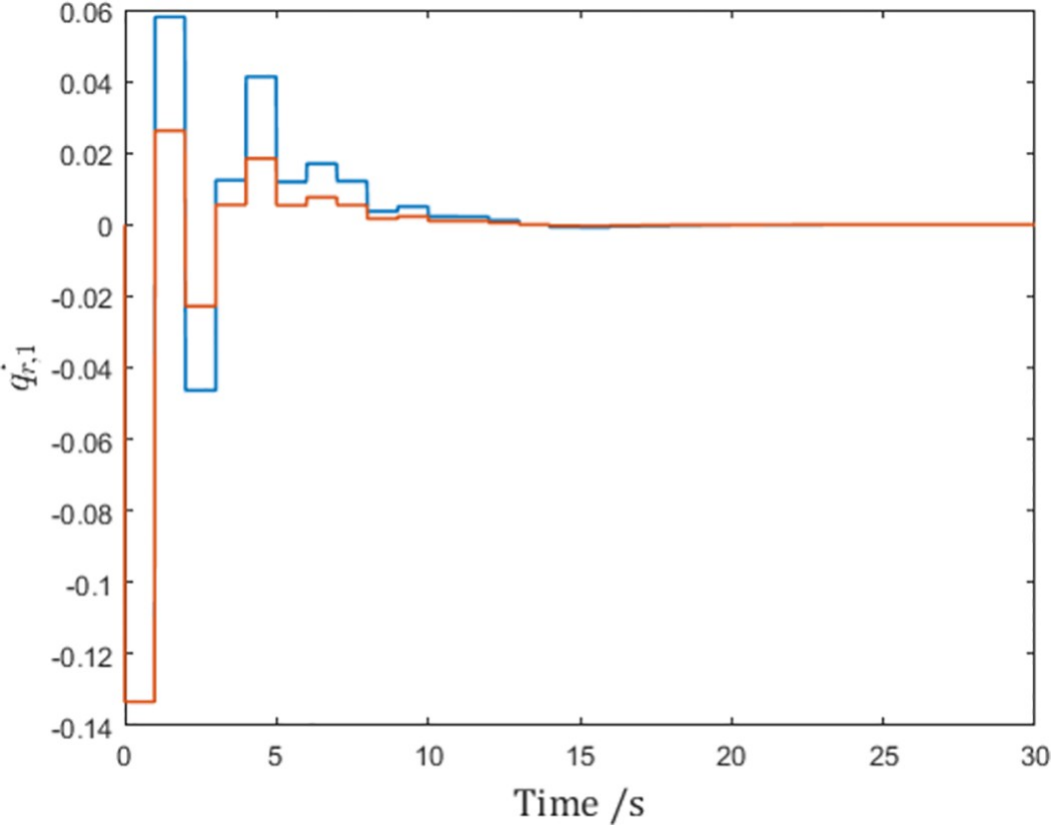
The reference velocity ${\dot{q}}_{r,1}$ of Agent 1.

As is shown by the vertical lines in the figures, although the generalized positions reach consensus as shown in Fig 3, the control input *τ*_1_ in Fig 4 becomes impulses at sampling instants due to the derivation of the reference velocity ${\dot{q}}_{r,1}$ in Fig 5. In fact, this issue affects agents. This means although the network reaches consensus in simulation, the actuators of the agent have to make impulsive infinities to achieve consensus, which is impossible in real-world scenarios.

### Simulation with the differentiable pulse function

In the proposed pulse function example, we use the same multi-agent system with the same sampled communication. The difference is the pulse function in the control law. Sinusoid functions are selected to meet the requirements (8) and (9). According to the consensus condition (16), the pulse function $\hat{\alpha }$ should be chosen such that

$$\overset{t_{k}+d_{k}}{\underset{t_{k}}{\int }}\alpha \left(\tau ,t_{k}\right)dt<\frac{2}{3}$$

**Fig 6:**
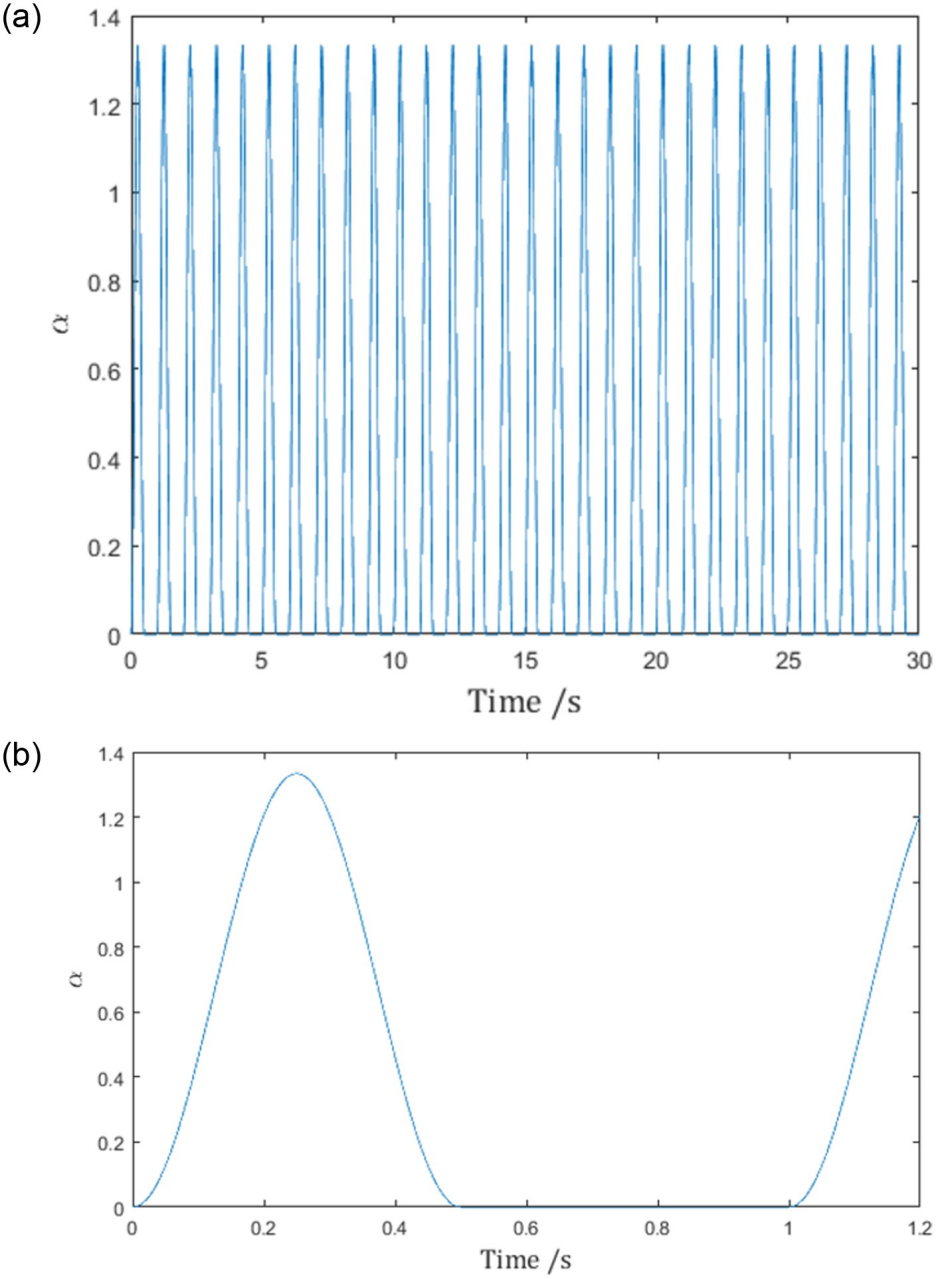
(a) The pulse function *α*(*t*). (b) the detailed view of *α*(*t*).

The sampling interval *h* = 1 and *d*_*k*_ = 0.5. Choose

$$\alpha \left(t,\;t_{k}\right)=\frac{2}{3}-\frac{2}{3}cos\left(\frac{t-t_{k}}{d_{k}}\cdot 2\pi \right)$$ (26)

so that the integral $\int_{t_{k}}^{t_{k}+d_{k}}\alpha \left(\tau ,t_{k}\right)dt=\frac{1}{3}$ and satisfies the consensus condition. And the resulting pulse function is shown in Fig 6(a), with more details in Fig 6(b):

The lower bound of the pulse function (26) is 0, and it fits the statement made in Remark 1: putting this bound into (24), we get $d\beta_{2}^{2}\rho {\left(Re\left(\lambda_{i}\right)+\left|Im\left(\lambda_{i}\right)\right|\right)}^{2}<0$, which makes (24) impossible to hold, i.e. the pulse function doesn’t satisfy (24), in other words, the consensus condition of [17] is incompatible with the pulse function.

**Fig 7:**
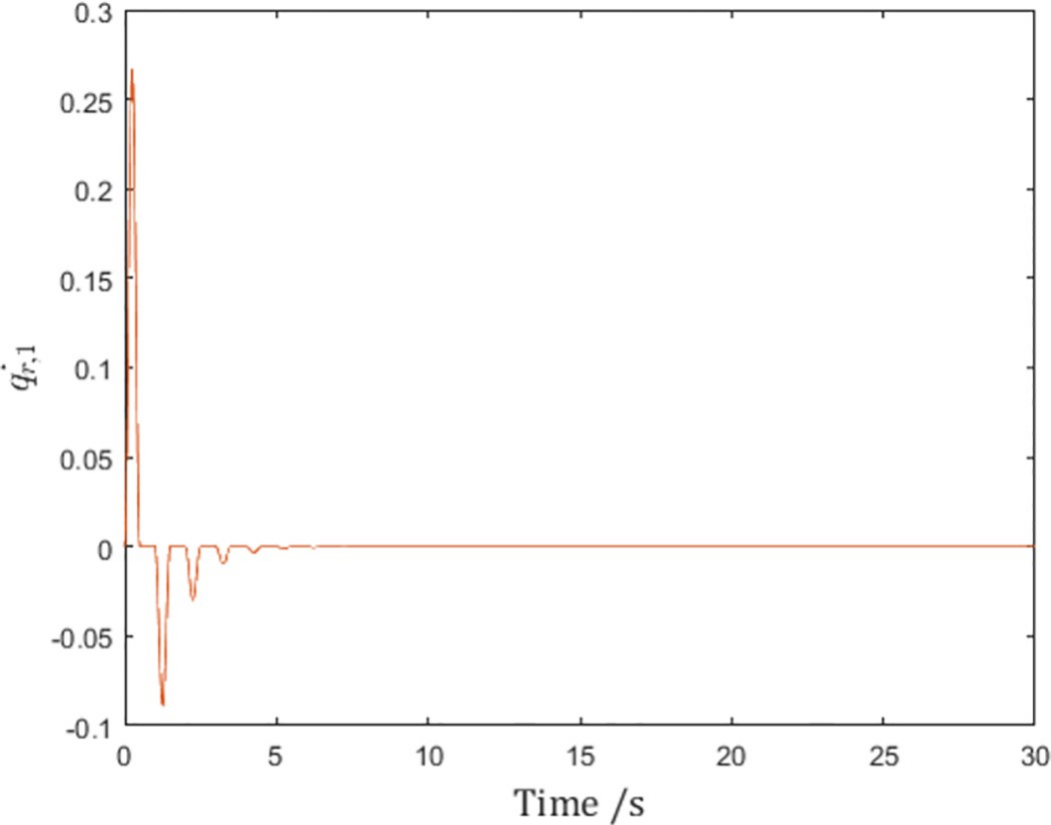
The reference velocity ${\dot{q}}_{r,1}$ of Agent 1 with the differentiable pulse function.

Since the chosen pulse function (26) satisfies the conditions in Lemma 1, it should be expected that the reference velocities ${\dot{q}}_{r,i}$ in Fig 7 are differentiable, and the control inputs *τ*_*i*_ are therefore finite.

**Fig 8:**
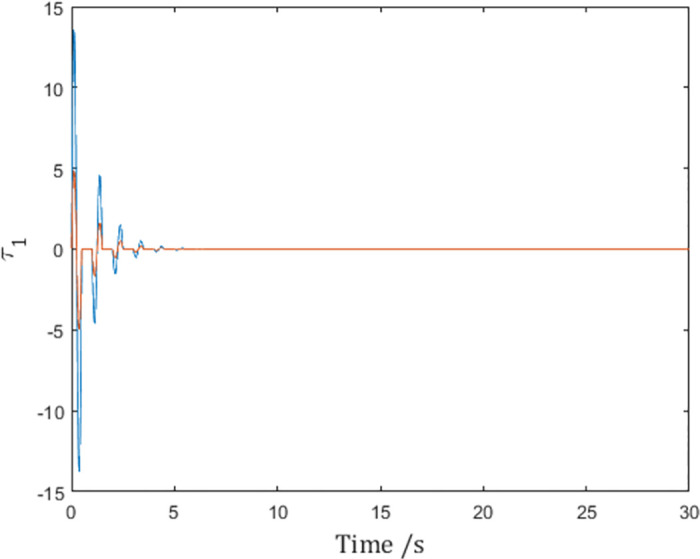
Control input *τ*_1_ of Agent 1 with the differentiable pulse function.

As is shown in Fig 8, the control input of Agent 1 is finite, and the infinity problem is solved.

**Fig 9:**
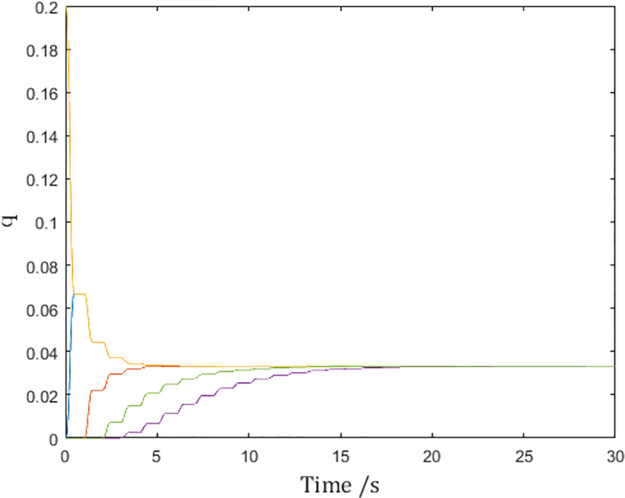
Generalized positions of the networked Euler-Lagrange systems.

And Fig 9 shows that the multi-agent system reaches consensus as calculated. On the surface, consensus seems also to be attained in the simulation without the pulse function, but it did so with infinite control input and is impossible in practice.

It can be inferred from (18) that consensus speed is only dependent on the sampling interval and the value of $\int_{t_{k}}^{t_{k+1}}\alpha \left(\tau ,\;t_{k}\right)d\tau$. A different *α*(*t*, *t*_*k*_) will not affect consensus speed when it has the same $\int_{t_{k}}^{t_{k+1}}\alpha \left(\tau ,\;t_{k}\right)d\tau$. Choose *d*_*k*_ = *h* = 1 and

$$\alpha \left(t,\;t_{k}\right)=\frac{1}{3}-\frac{1}{3}cos\left(\frac{t-t_{k}}{d_{k}}\cdot 2\pi \right)$$

**Fig 10:**
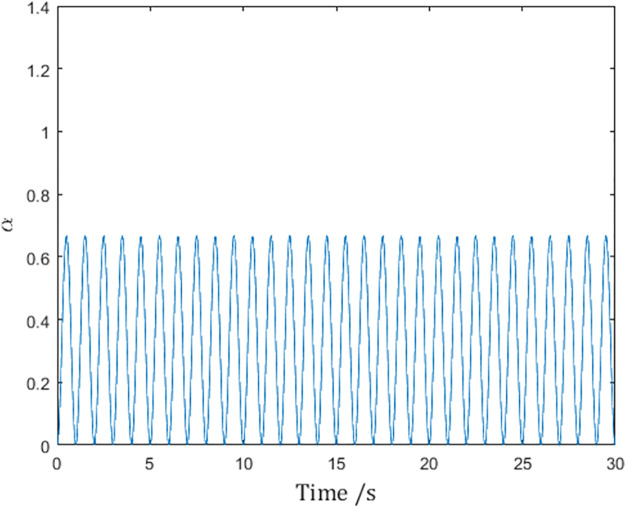
The pulse function *α*(*t*).

The resulting pulse function is shown in Fig 10, and $\int_{t_{k}}^{t_{k}+d_{k}}\alpha \left(\tau ,t_{k}\right)dt=\frac{1}{3}$ is unchanged, we get

**Fig 11:**
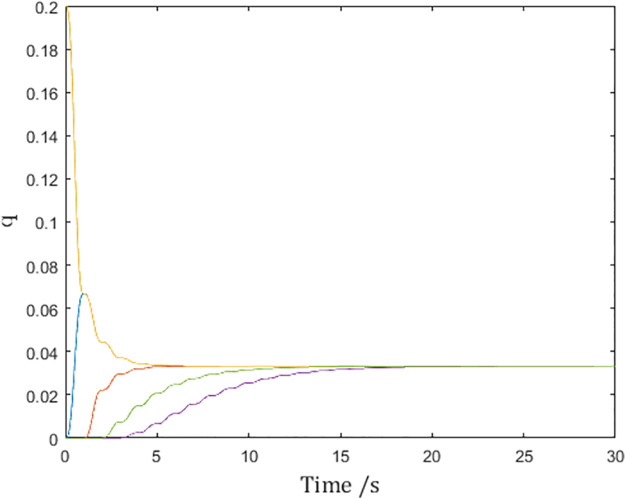
Generalized positions of the networked Euler-Lagrange systems.

Fig 11 shows the same consensus speed as in Fig 9, confirming the inference.

**Fig 12:**
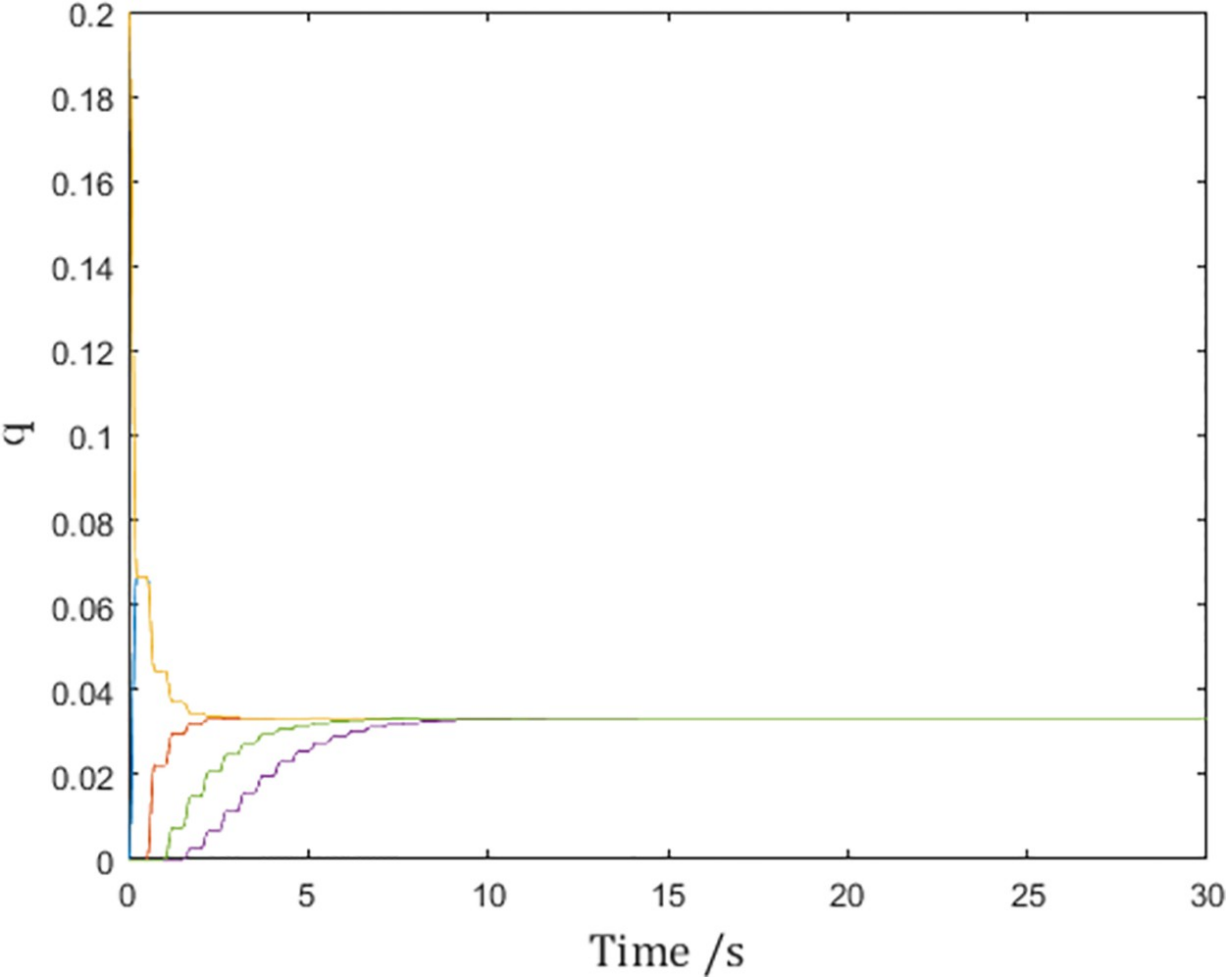
Generalized positions of the networked Euler-Lagrange systems.

**Fig 13:**
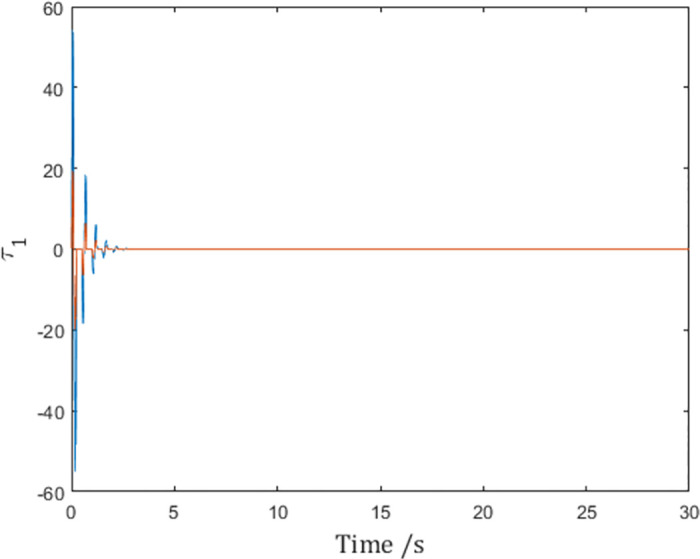
Control input *τ*_1_ of Agent 1.

Consensus speed can also be tuned independently while maintaining condition (16). For example, set the sampling interval to 0.5 and *d*_*k*_ = 0.25, and choose

$$\alpha \left(t,\;t_{k}\right)=\frac{4}{3}-\frac{4}{3}cos\left(\frac{t-t_{k}}{d_{k}}\cdot 2\pi \right)$$

to preserve the value of $\int_{t_{k}}^{t_{k}+d_{k}}\alpha \left(\tau ,t_{k}\right)dt$. The effect is that the consensus speed is doubled as shown in Fig 12:

but the negative effect is that the value of ${\ddot{q}}_{r,i}$ is quadrupled as shown in Fig 13, putting more stress on the controller, as is shown in Fig 8. In fact, when the value of $\int_{t_{k}}^{t_{k}+d_{k}}\alpha \left(\tau ,t_{k}\right)dt$ is chosen, the only means to achieve faster consensus is to select a narrow and tall shaped *α*(*t*, *t*_*k*_), which inevitably leads to larger $\dot{\alpha }\left(t,t_{k}\right)$ and hence larger ${\ddot{q}}_{r,i}$.

## Conclusion

This paper reviews existing work on the sampled-data consensus of networked Euler-Lagrange systems. To address its flaw that steers the control inputs to infinity, a new controller with specially designed differentiable pulse functions is designed. It is proved that the networked Euler-Lagrange systems can reach consensus when a criterion on the pulse function is satisfied. Finally, numerical simulations are given to verify the theoretical results.

## Supporting information

S1 Fig The geometric interpretation. (PDF)

S2 Fig The communication graph. (PDF)

S3 FigThe reference velocity ${\dot{q}}_{r,1}$ of Agent 1.(PDF)

S4 Fig The control input τ_1_ of Agent 1. (PDF)

S5 Fig The generalized positions of the system. (PDF)

S6 Fig The pulse function α(t). (PDF)

S7 Fig The detailed view of α(t). (PDF)

S8 FigThe reference velocity ${\dot{q}}_{r,1}$ of Agent 1 with the differentiable pulse function.(PDF)

S9 Fig Control input τ_1_ of Agent 1 with the differentiable pulse function. (PDF)

S10 Fig Generalized positions of the networked Euler-Lagrange systems. (PDF)

S11 Fig The pulse function α(t). (PDF)

S12 Fig Generalized positions of the networked Euler-Lagrange systems. (PDF)

S13 Fig Generalized positions of the networked Euler-Lagrange systems. (PDF)

S14 Fig Control input τ_1_ of Agent 1. (PDF)
