# Supplementary figures and images for: Modulated sampled-data consensus for networked Euler-Lagrange systems with differentiable pulse function
Source: PLoS One. 2022 Nov 10;17(11):e0274461. doi: 10.1371/journal.pone.0274461 (PMC9648764; doi:10.1371/journal.pone.0274461)

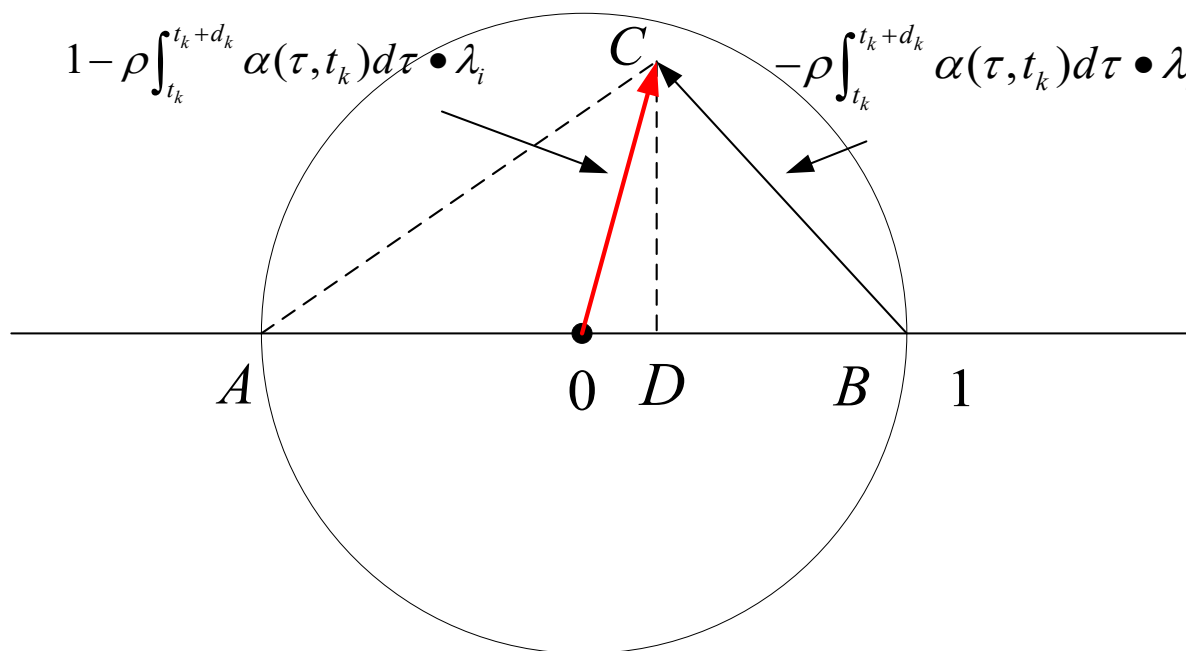

Supplement: S1 Fig — (PDF) [file pone.0274461.s001.pdf]

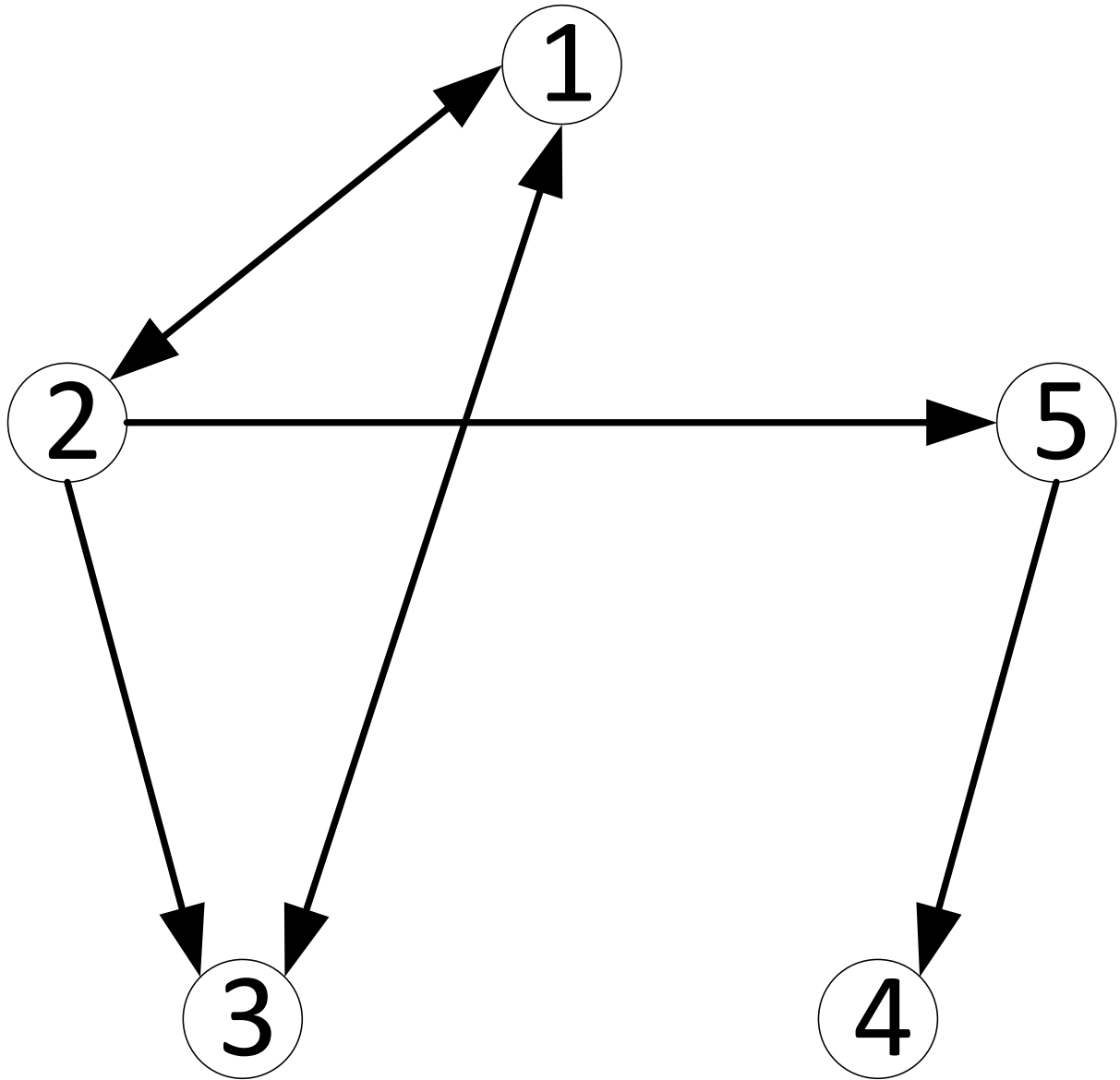

Supplement: S2 Fig — (PDF) [file pone.0274461.s002.pdf]

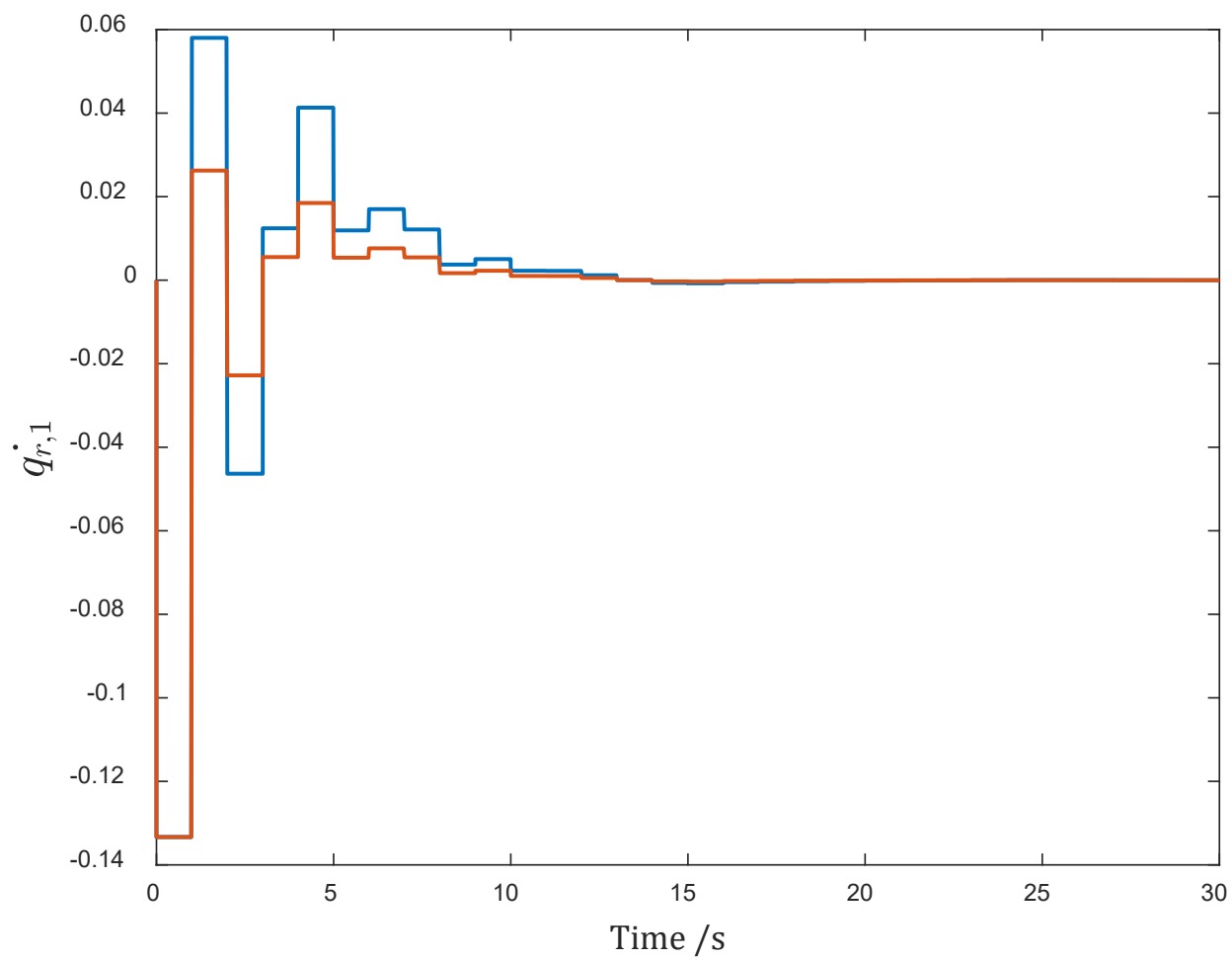

Supplement: S3 Fig — (PDF) [file pone.0274461.s003.pdf]

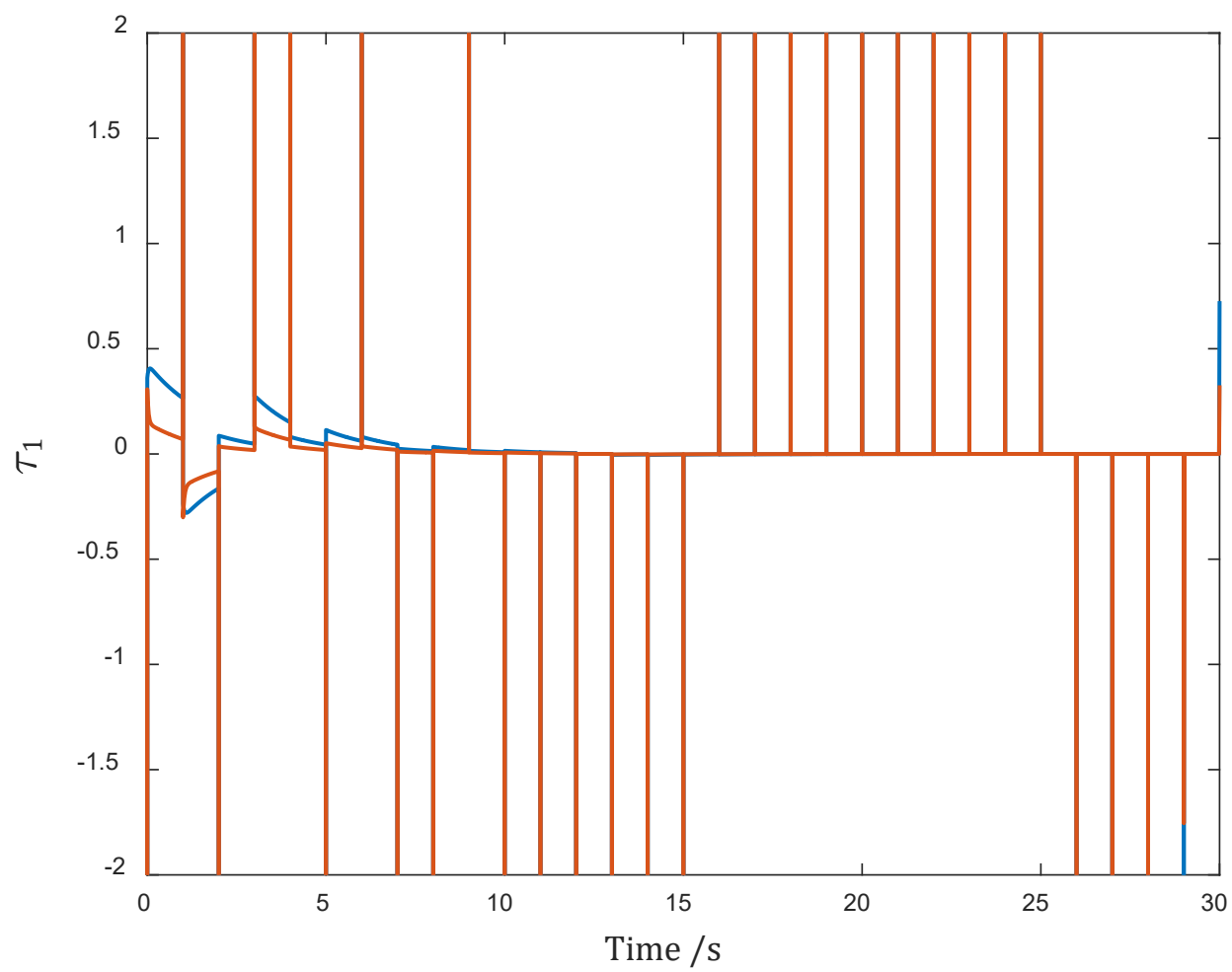

Supplement: S4 Fig — (PDF) [file pone.0274461.s004.pdf]

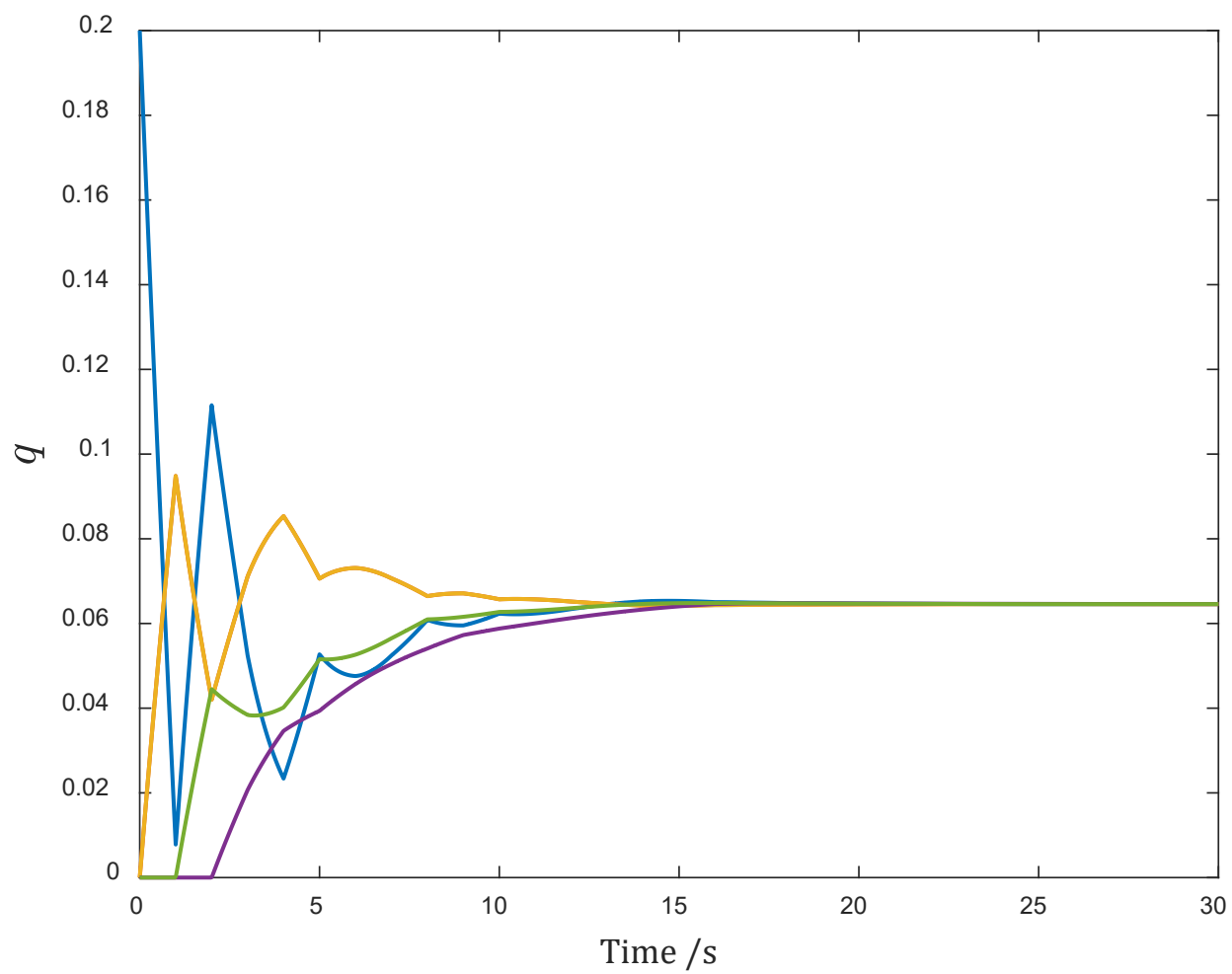

Supplement: S5 Fig — (PDF) [file pone.0274461.s005.pdf]

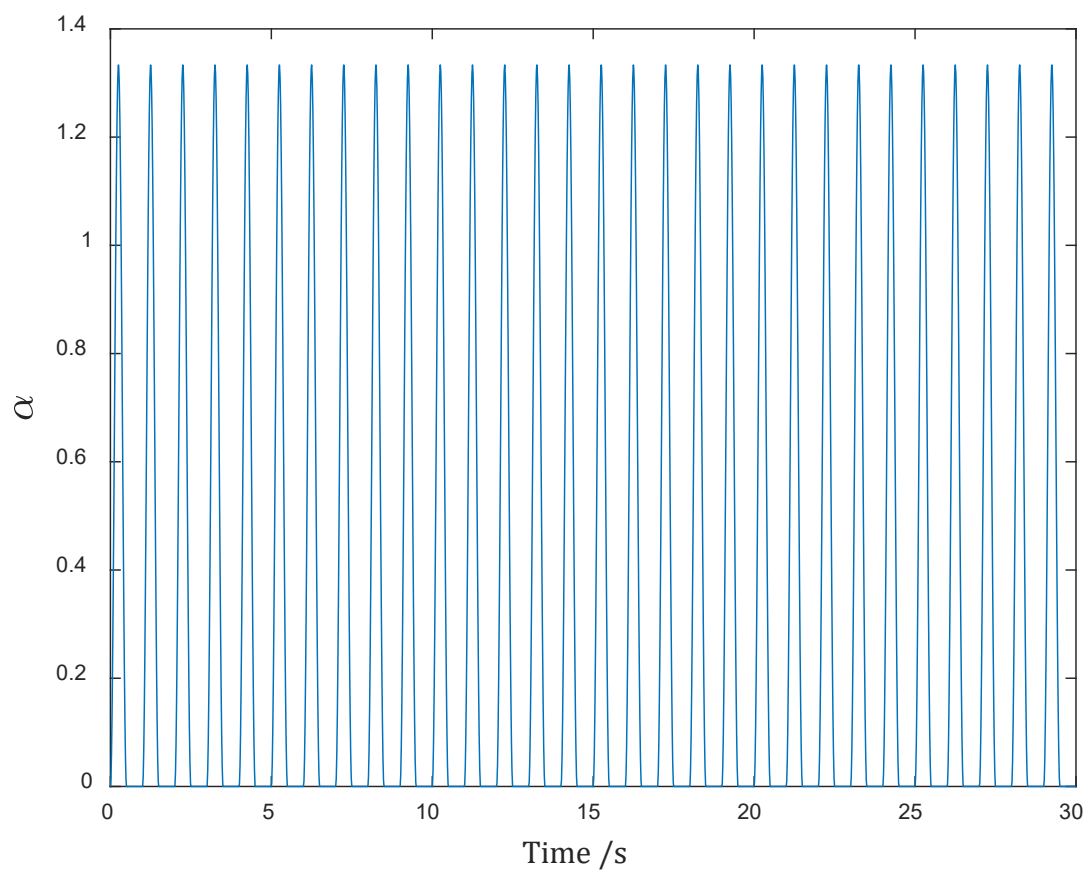

Supplement: S6 Fig — (PDF) [file pone.0274461.s006.pdf]

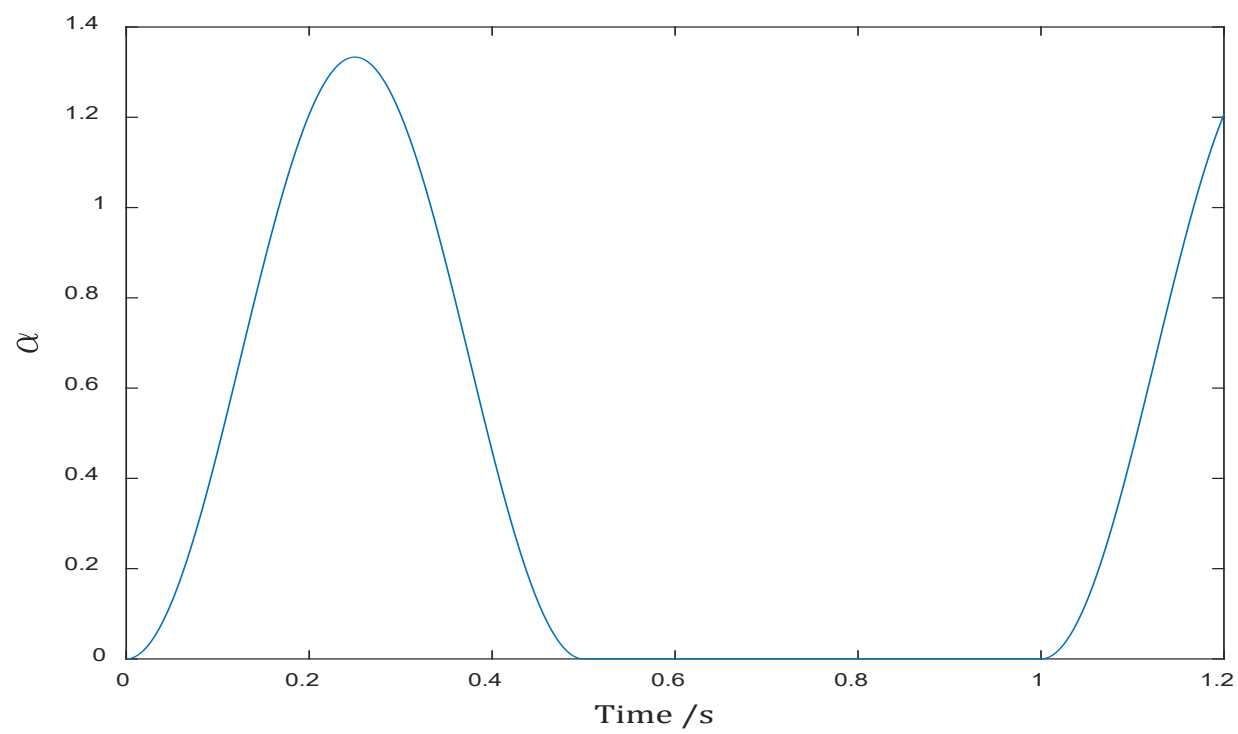

Supplement: S7 Fig — (PDF) [file pone.0274461.s007.pdf]

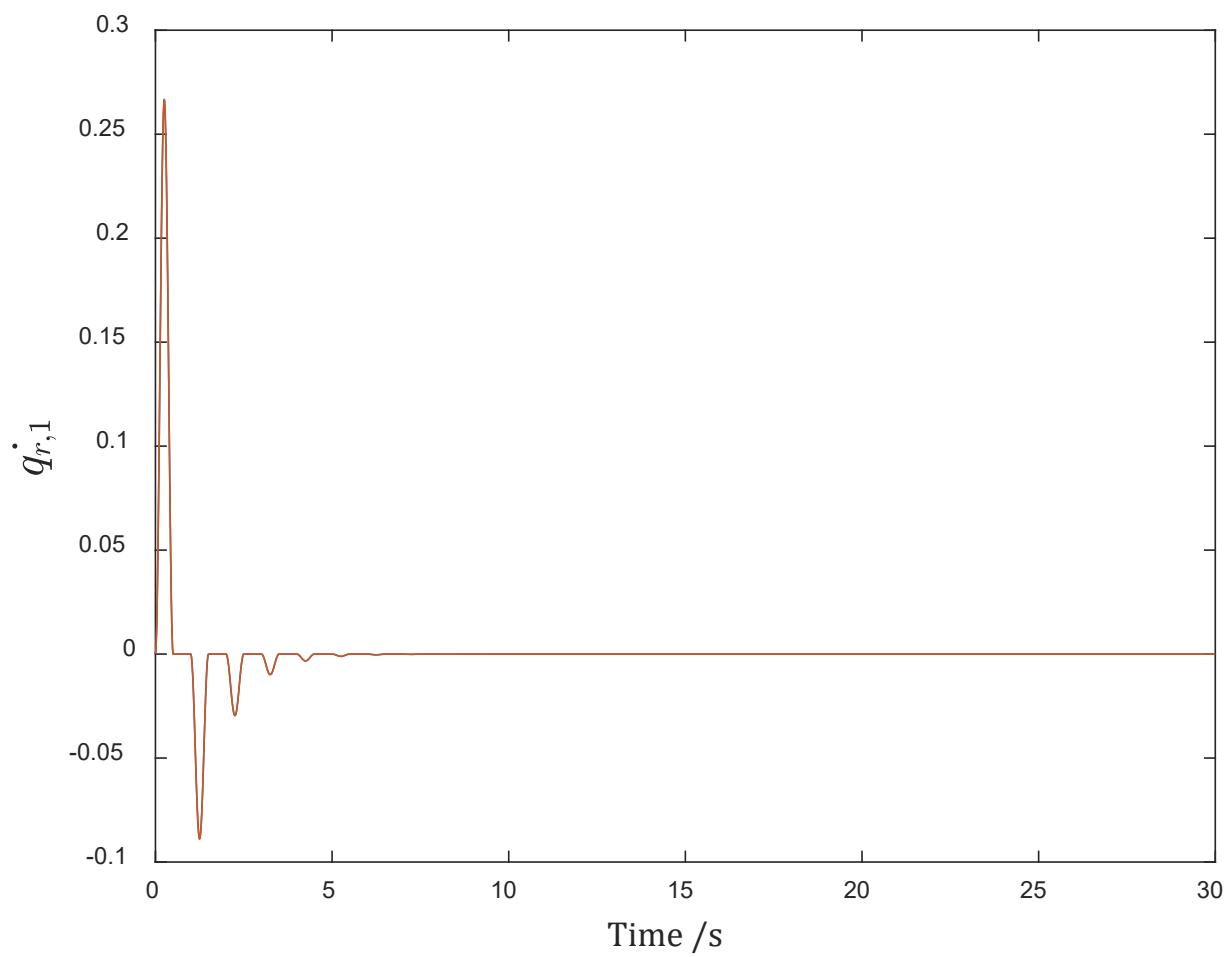

Supplement: S8 Fig — (PDF) [file pone.0274461.s008.pdf]

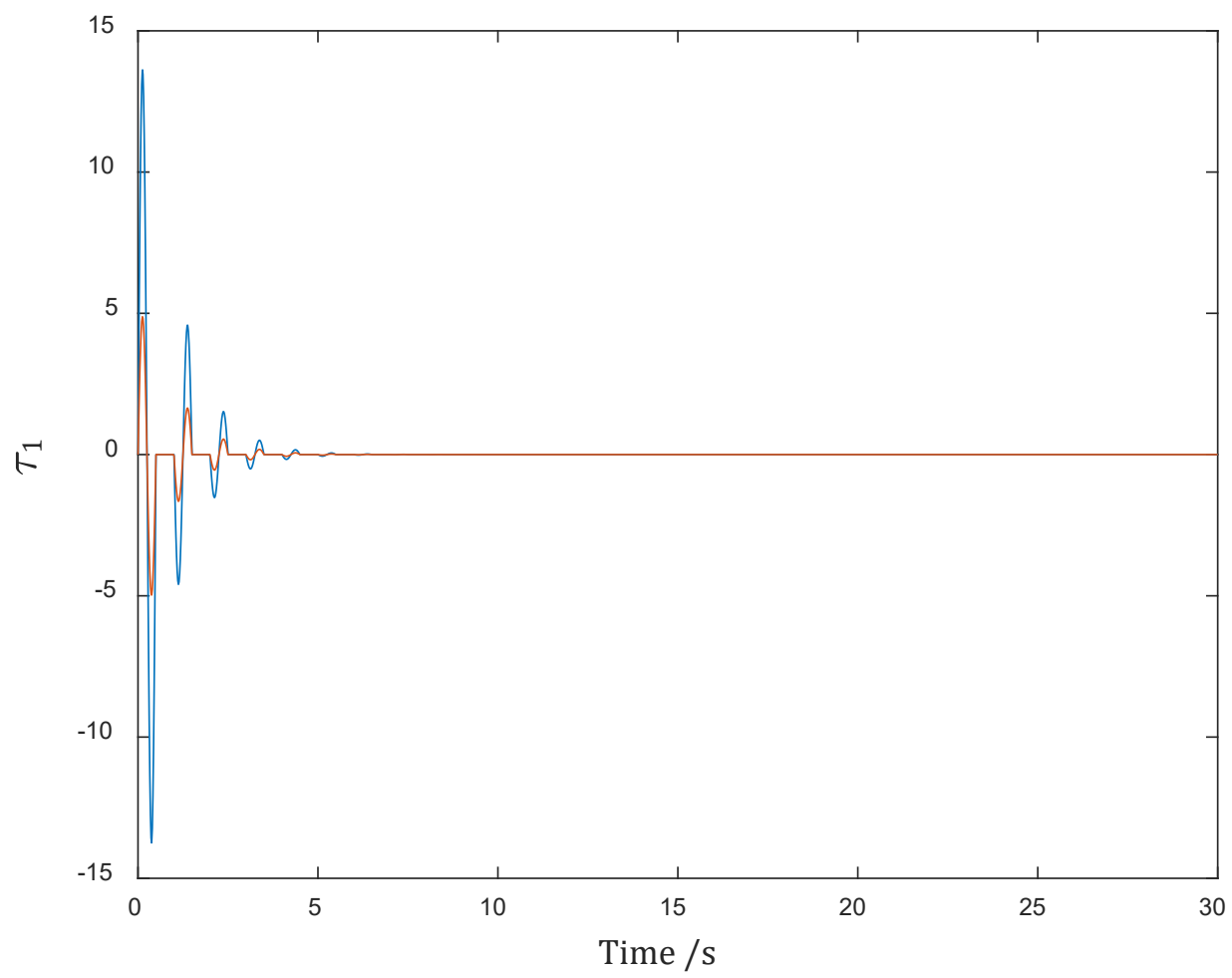

Supplement: S9 Fig — (PDF) [file pone.0274461.s009.pdf]

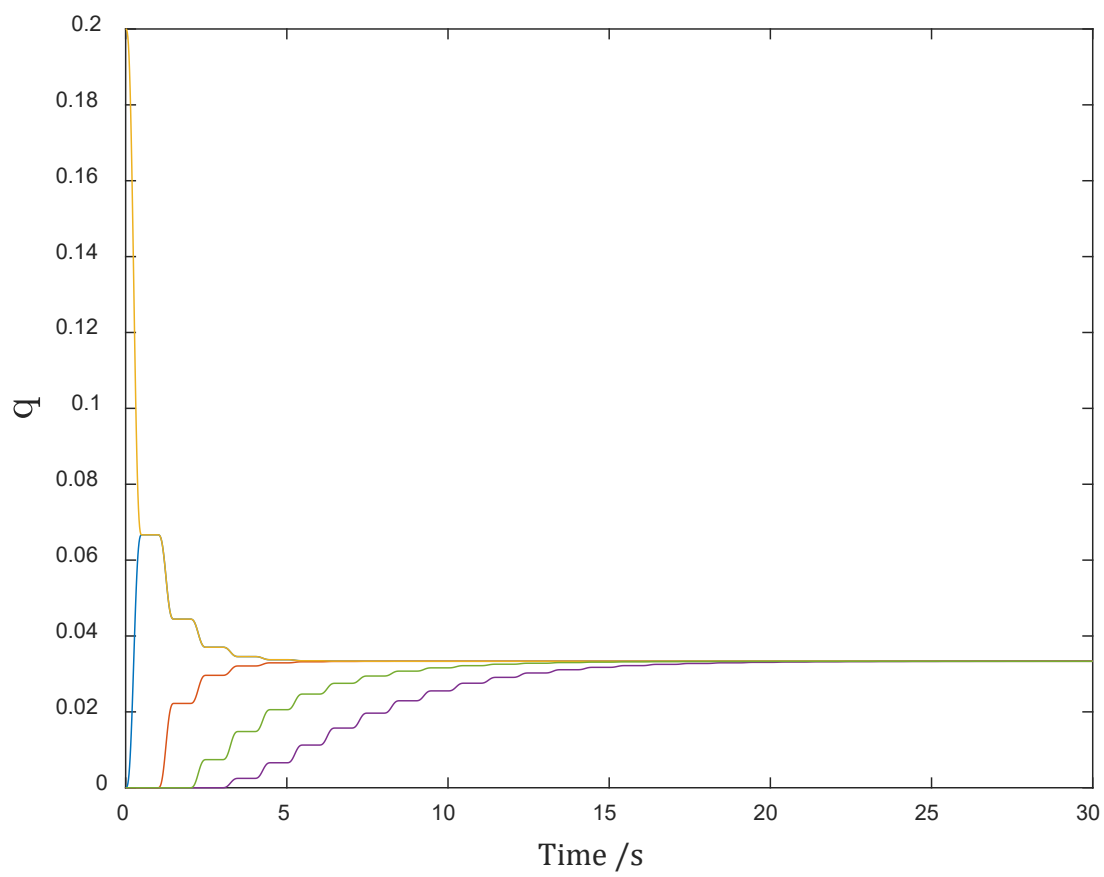

Supplement: S10 Fig — (PDF) [file pone.0274461.s010.pdf]

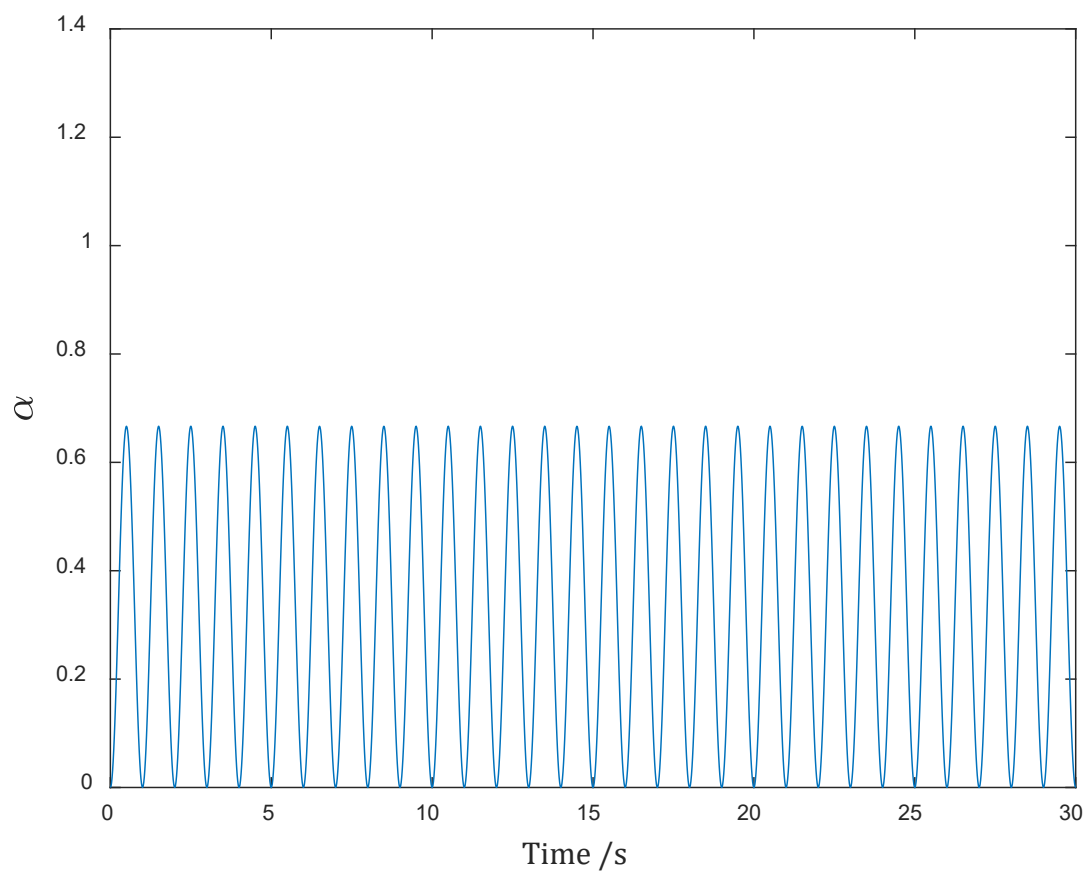

Supplement: S11 Fig — (PDF) [file pone.0274461.s011.pdf]

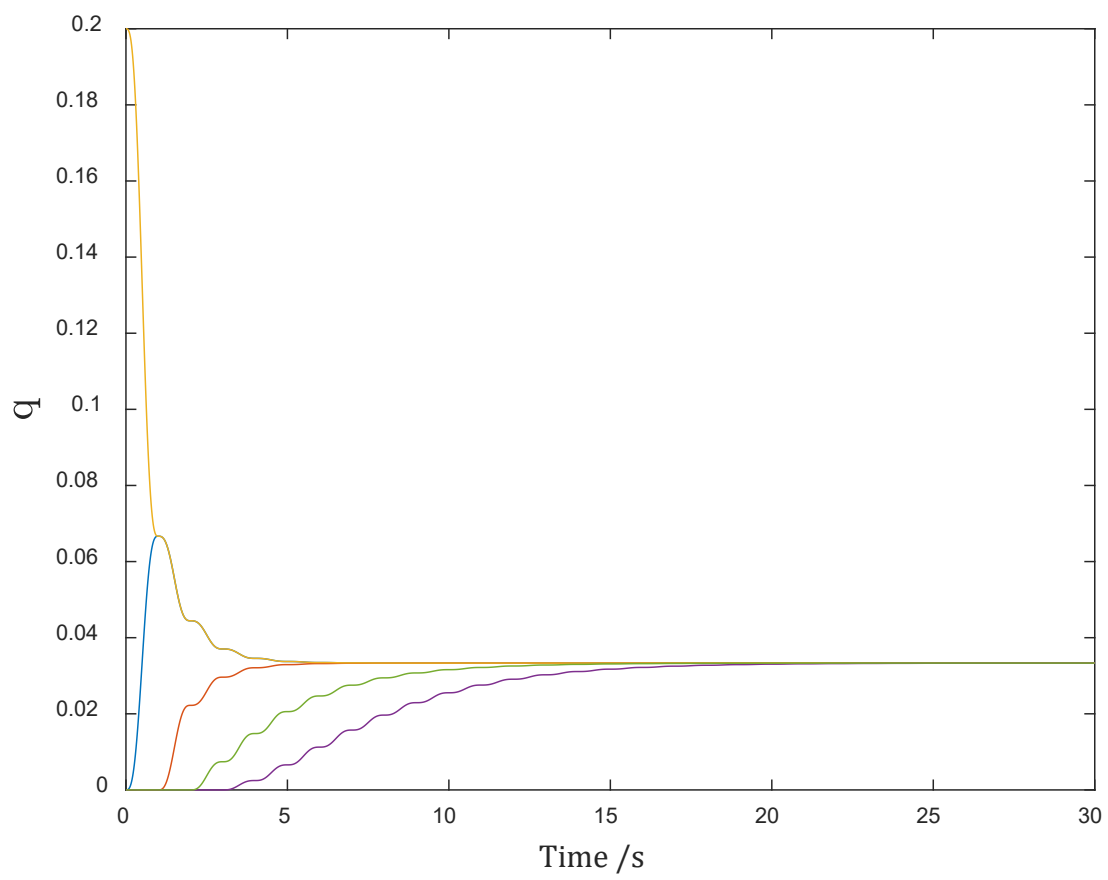

Supplement: S12 Fig — (PDF) [file pone.0274461.s012.pdf]

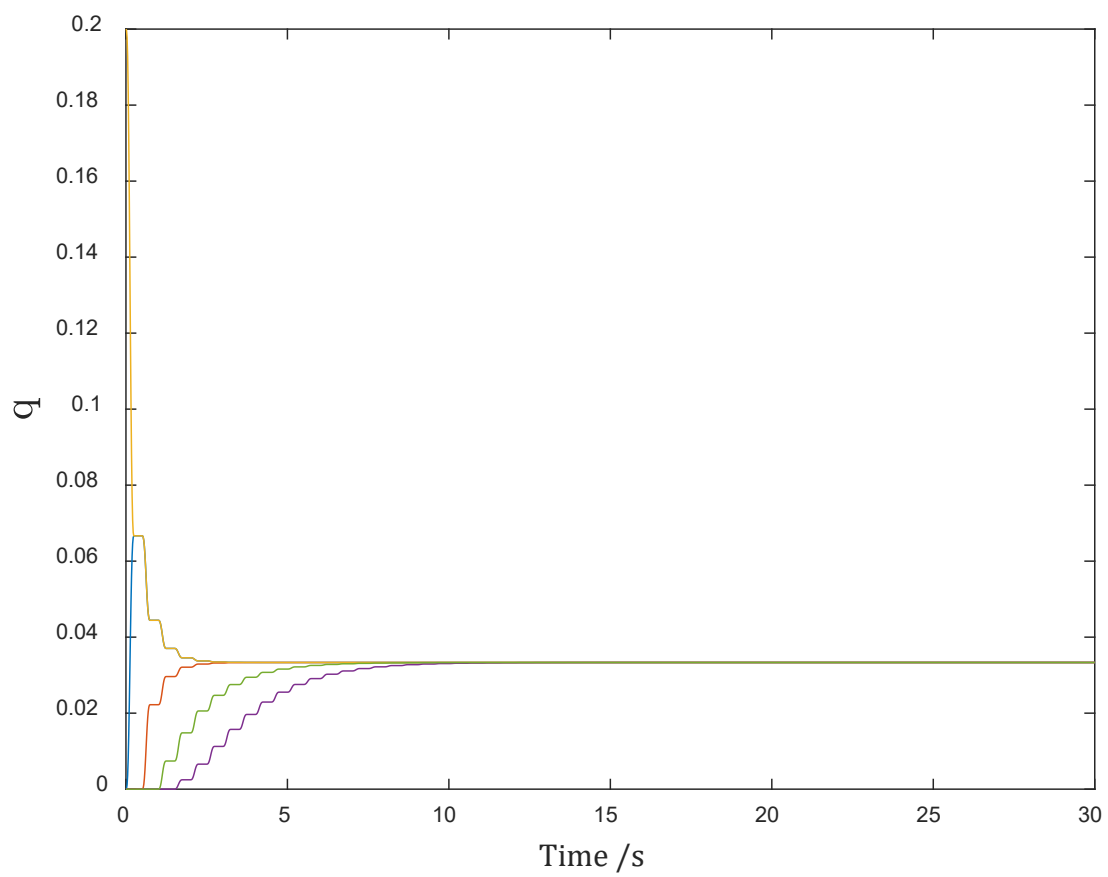

Supplement: S13 Fig — (PDF) [file pone.0274461.s013.pdf]

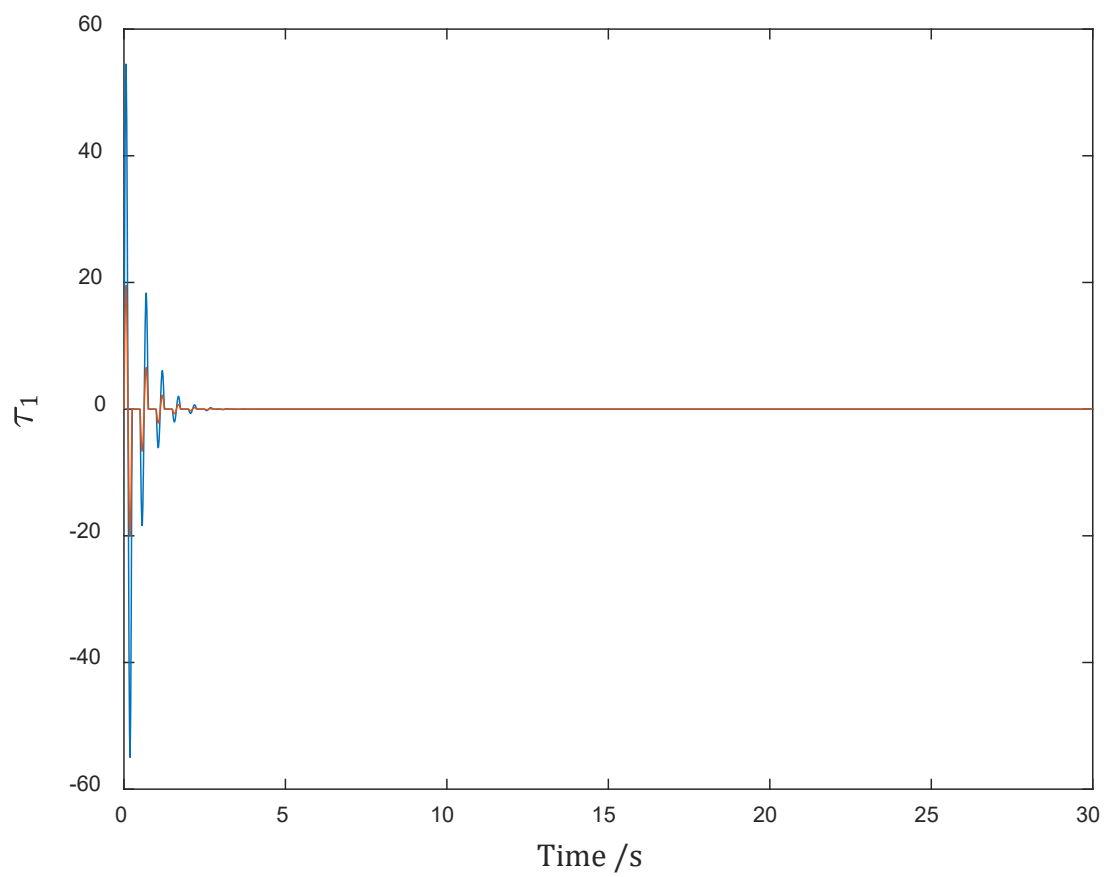

Supplement: S14 Fig — (PDF) [file pone.0274461.s014.pdf]
